# Supplementary material for: Atomic reconstruction for realizing stable solar-driven reversible hydrogen storage of magnesium hydride
Source: Nat Commun. 2024 Apr 1;15:2815. doi: 10.1038/s41467-024-47077-y (PMC10984991; doi:10.1038/s41467-024-47077-y)
Supplement: Supplementary file 1 — Supplementary Information [file 41467_2024_47077_MOESM1_ESM.pdf]

Supplementary Information

Atomic Reconstruction for Realizing Stable Solar-Driven Reversible Hydrogen Storage of Magnesium Hydride

Xiaoyue Zhang[1], Shunlong Ju[1], Chaoqun Li[1], Jiazheng Hao[2,3], Yahui Sun[1], Xuechun Hu[1], Wei Chen[1], Jie Chen[2,3], Lunhua He[2,4,5], Guanglin Xia[1,*], Fang Fang[1,*], Dalin Sun[1], Xuebin Yu[1,*]

[1]Department of Materials Science, Fudan University, Shanghai 200433, China

[2]Spallation Neutron Source Science Center, Dongguan 523803, China

[3]Institute of High Energy Physics, Chinese Academy of Sciences, Beijing 100049, China

[4]Beijing National Laboratory for Condensed Matter Physics, Institute of Physics, Chinese Academy of Sciences, Beijing 100190, PR China

[5]Songshan Lake Materials Laboratory, Dongguan 523808, PR China

*Email: xiaguanglin@fudan.edu.cn; f_fang@fudan.edu.cn; yuxuebin@fudan.edu.cn

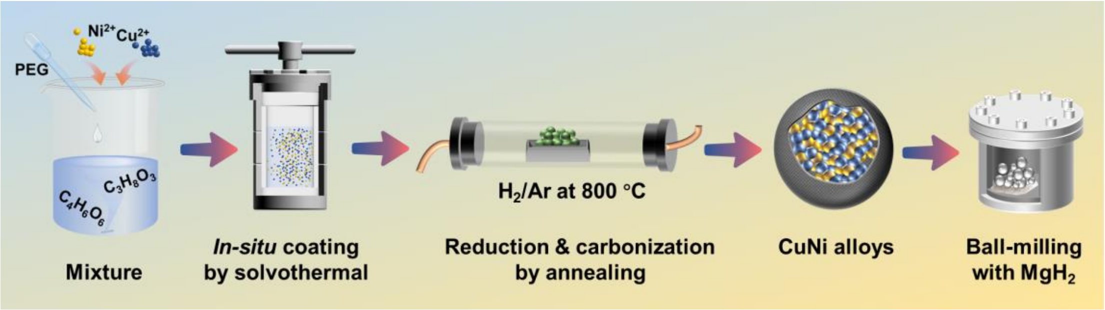

Supplementary Figure 1. Preparation process of the samples. Schematic illustration of the fabrication of CuNi alloys and composite with MgH_2.

Supplementary Table 1. Elemental analysis of the CuNi alloys.

Sample name	Molar ratio of Cu and Ni salts	Composition (Cu:Ni) (w/w) [a]	Atomic ratio
Cu_1Ni_1	1:1	46.51: 43.88	1: 1.03
Cu_1Ni_2	1:2	31.56: 58.22	1: 2.01
Cu_1Ni_3	1:3	23.89: 64.48	1: 3.01

[a] Composition is measured by ICP-OES technique.

Supplementary Table 2. XPS surface elemental analysis of Cu_1Ni_1.

Element	Atomic %	Element	Atomic %
Cu	16.37	C	60.10
Ni	15.72	O	3.98

The carbon layer accounted for 60.1% of the element analysis due to the wrapping of alloys, but the proportion of O is much lower than that of other elements, indicating that the gas-phase reduction method effectively controlled the oxidation of alloys.

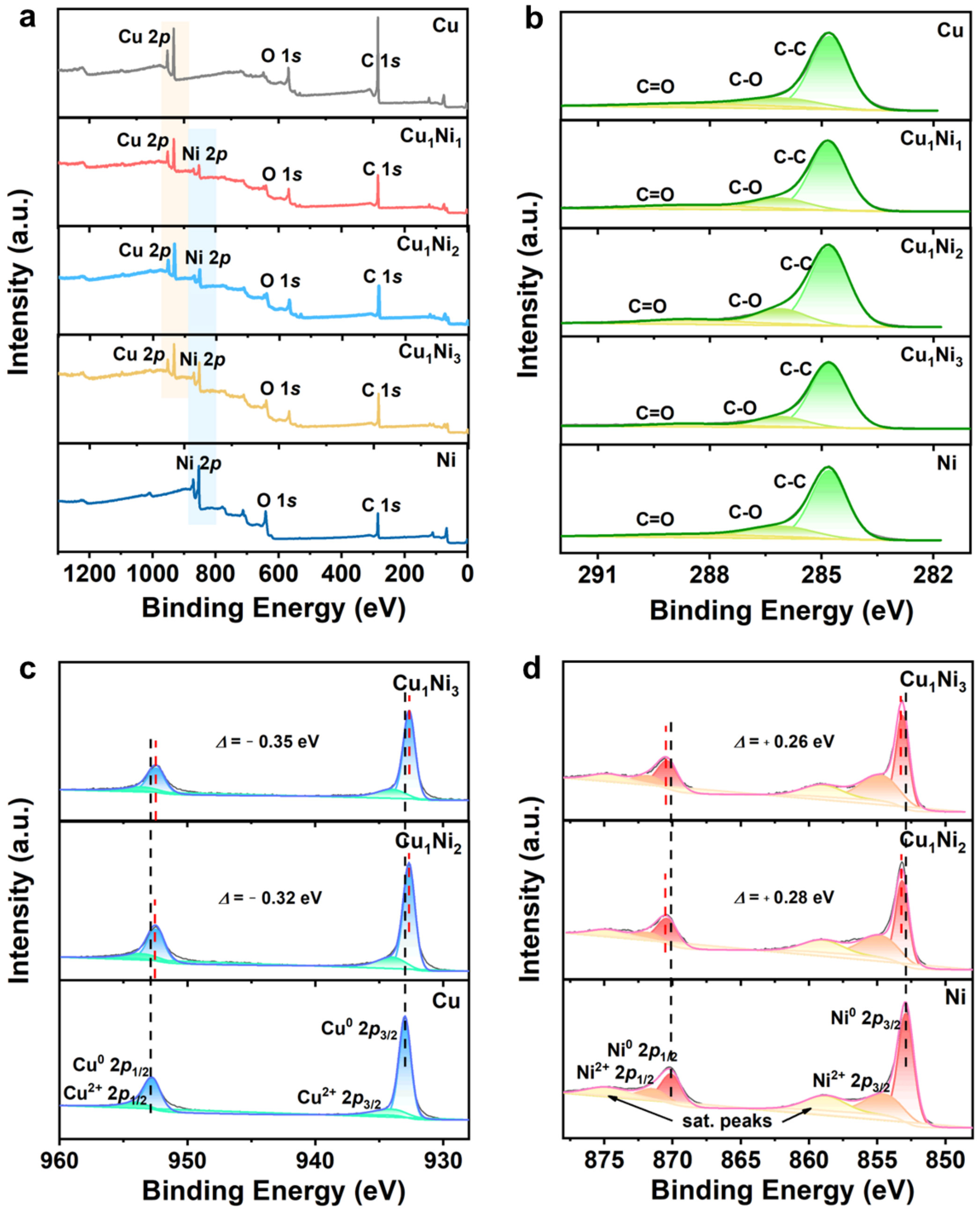

Supplementary Figure 2. XPS survey spectra of CuNi alloys. a) XPS survey spectra and b) high-resolution C 1*s* XPS spectrum of CuNi alloys, including Cu and Ni for comparison. High-resolution c) Cu *2p* and d) Ni *2p* XPS spectrum of Cu_1Ni_2 and Cu_1Ni_3, including Cu and Ni for comparison. The unit a.u. means to arbitrary units. Source data are provided as a source data file.

The C 1*s* spectrum showed fitting peaks at 284.8, 286.1, and 288.6 eV, corresponding to C-C, C-O, and C=O, indicating no chemical interaction between the outer wrapped carbon layer and CuNi alloys.

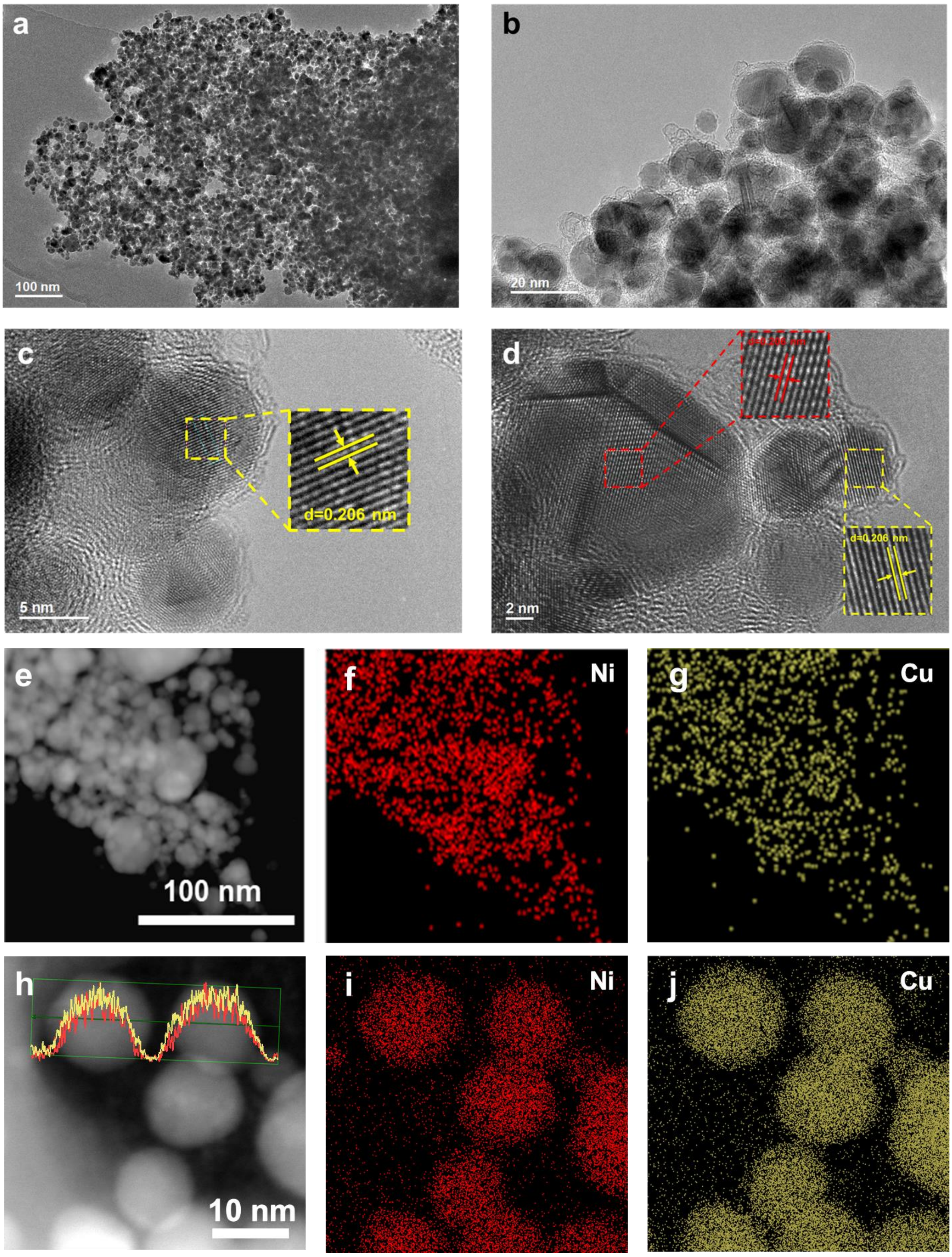

Supplementary Figure 3. TEM measurements of Cu_1Ni_1 alloys. a-d) TEM images of Cu_1Ni_1 alloys. e-j) EDS elemental mapping images with line scanning of Cu_1Ni_1 alloys.

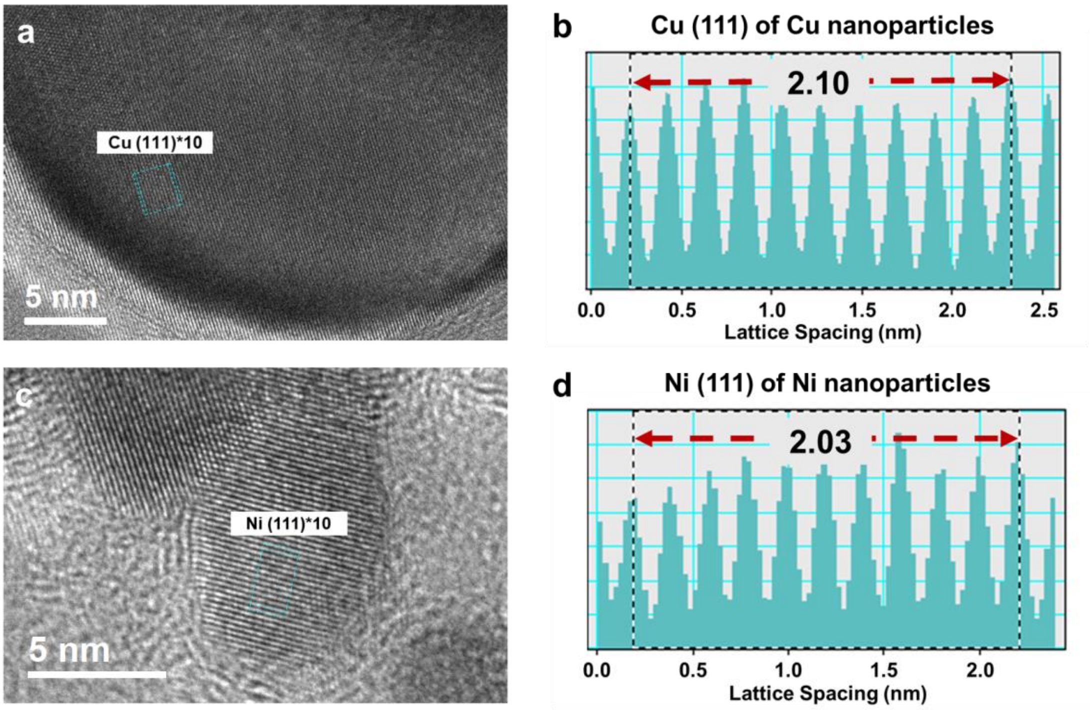

Supplementary Figure 4. TEM measurements of Cu and Ni nanoparticles. TEM images and lattice spacing of a-b) Cu and c-d) Ni nanoparticles.

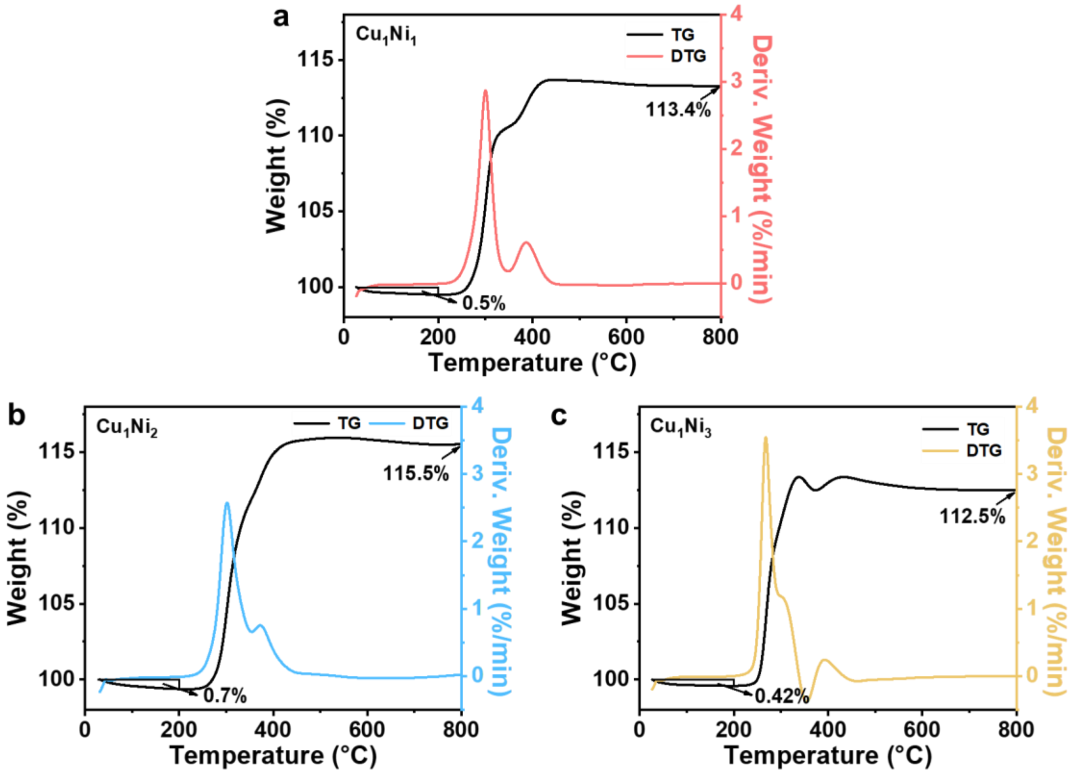

Supplementary Figure 5. Carbon content analysis. Thermogravimetric analysis (TGA) of a) Cu_1Ni_1, b) Cu_1Ni_2, and c) Cu_1Ni_3 alloys. Source data are provided as a source data file.

Based on TGA results of Cu_1Ni_1, the proportion of metal and carbon in the alloy could be roughly analyzed. TGA was conducted at a heating rate of 10 °C min^{-1} in air. As the temperature increased from 200 °C to 700 °C, the alloys' mass continued to increase due to the oxidation of CuNi alloy in the air. The rate of increase in material mass slowed down significantly between 300 °C and 400 °C, caused by the loss of the outer carbon layer due to oxidation. At 800 °C, the material's mass was 113.4% of the initial sample, and no further changes occurred. At this point, the carbon layer was completely lost, and CuNi alloy was fully oxidized to CuO and NiO, with a molar ratio of 1:1. The moisture content in the test sample was 0.5%. Based on this data, it could be inferred that the content of CuNi metal in the sample is 90.6%, and the carbon content is 8.9%.

Similarly, it could be inferred that the content of CuNi metal in $CuNi_2$ and Cu_1Ni_3 alloy is 91% and 89%, respectively, and the carbon content is 8.4% and 10.6%, respectively.

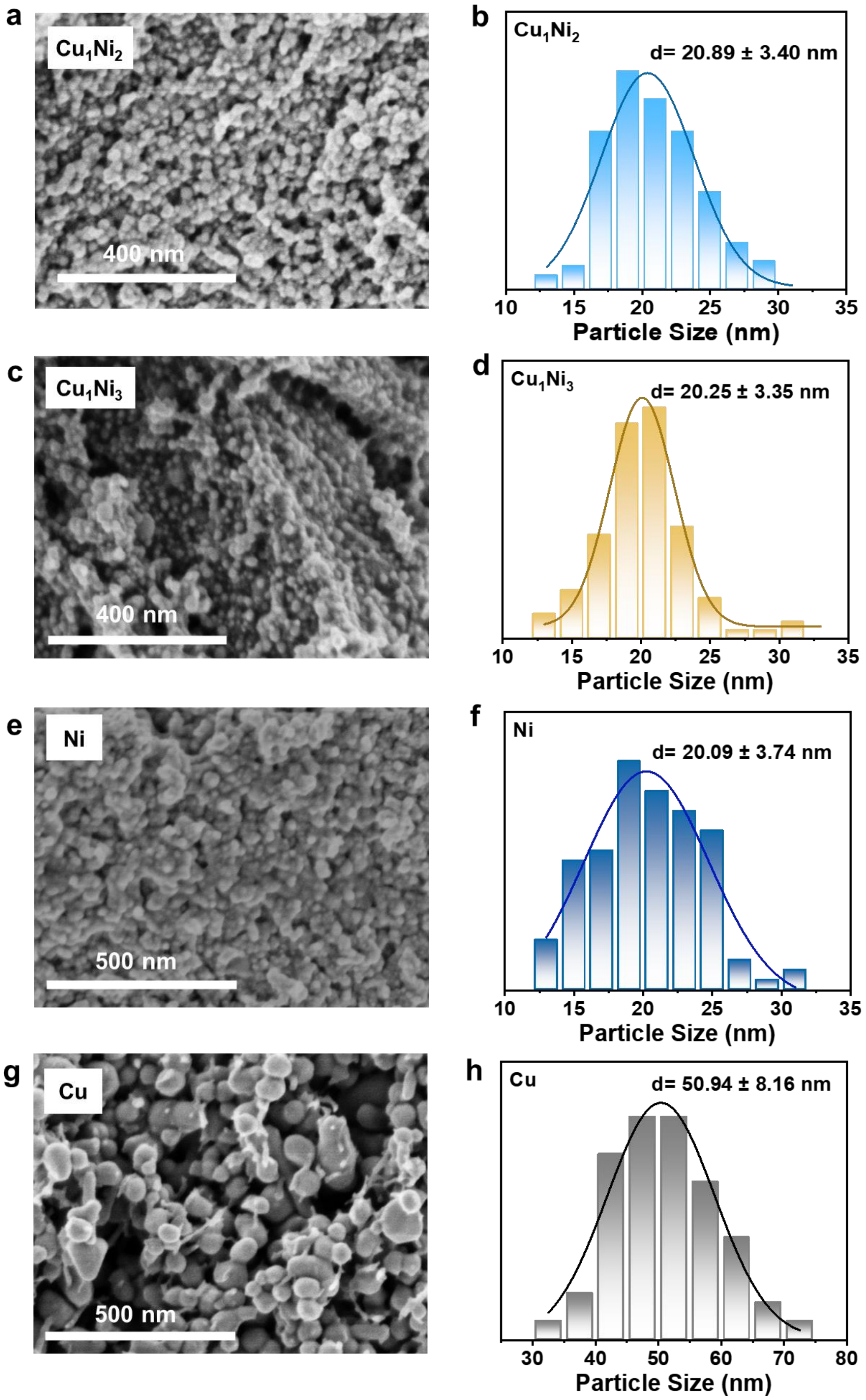

Supplementary Figure 6. Morphology and particle size of CuNi alloys. SEM images and the corresponding particle size distributions with Gaussian fitting of a-b) Cu_1Ni_2, c-d) Cu_1Ni_3, e-f) Ni, and g-h) Cu. Source data are provided as a source data file.

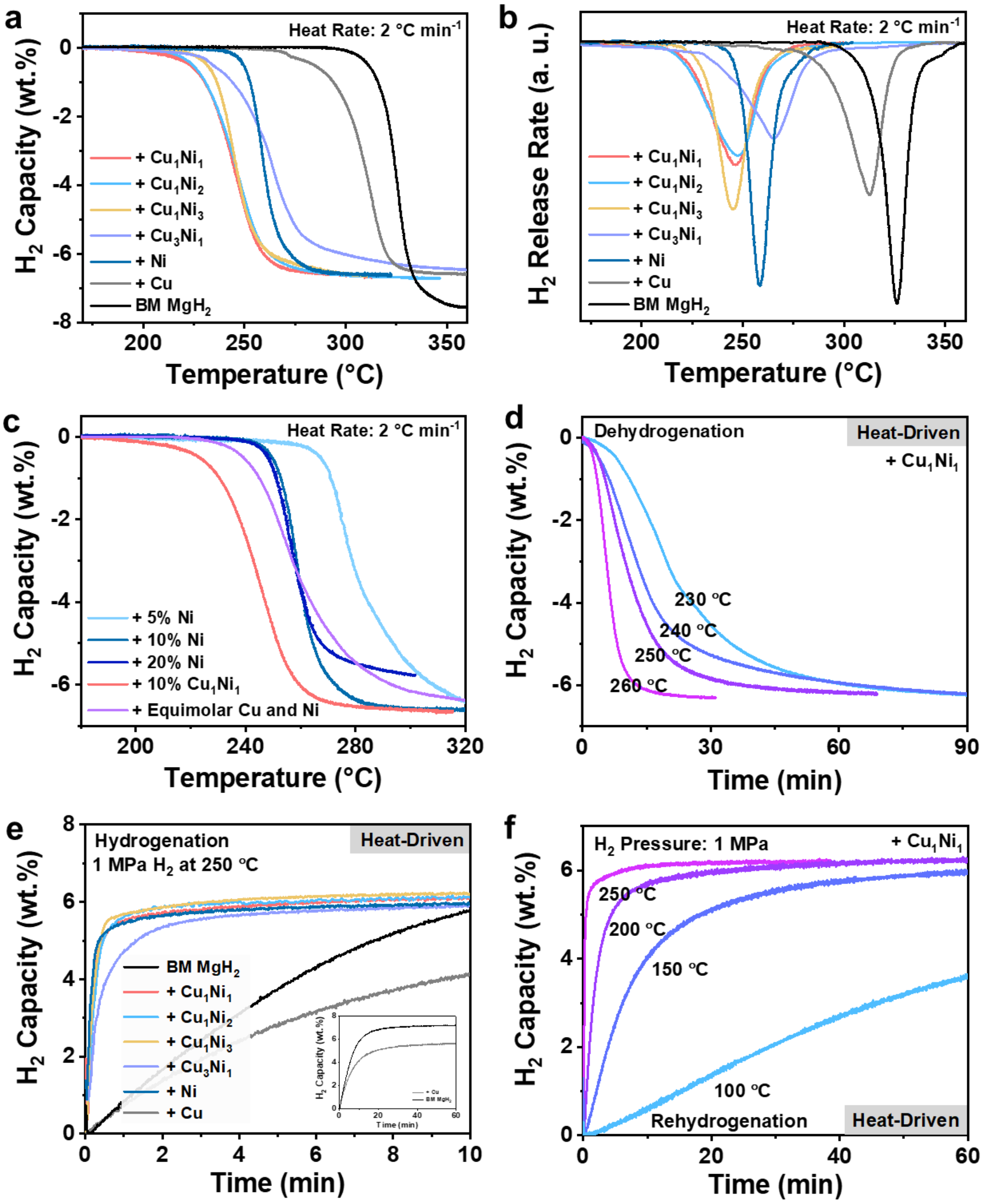

Supplementary Figure 7. Heat-driven temperature-programmed hydrogen desorption and isothermal hydrogenation performance. a) TPD curves and b) TPD derivative curves of MgH_2 under the catalysis of Cu_1Ni_1, Cu_1Ni_2, Cu_1Ni_3, Cu_3Ni_1, Ni, and Cu, including pure MgH_2 for comparison. The unit a.u. means to arbitrary units. c) TPD curves of MgH_2 under the catalysis of Ni with varying ratios, including MgH_2 catalyzed by 10% CuNi and equimolar Cu and Ni for comparison. d) Isothermal dehydrogenation profiles of MgH_2 under the catalysis of Cu_1Ni_1 at varying temperatures. e) Isothermal hydrogenation curves at 250 °C under 1 MPa of MgH_2 under the catalysis of Cu_1Ni_1, Cu_1Ni_2, Cu_1Ni_3, Cu_3Ni_1, Ni, and Cu, respectively, including ball-milled MgH_2 for comparison. f) Isothermal hydrogenation curves of MgH_2 under the catalysis of Cu_1Ni_1 at varying temperatures. Source data are provided as a source data file.

A series of catalytic performance of MgH_2 under the catalysis of CuNi alloys with

different components is first presented and analyzed, which provides a basis on the selection of CuNi alloys with suitable proportions for the subsequent solar-driven experiments.

Temperature-programmed desorption (TPD) curves and their differential curves (Supplementary Figure 7a-b) show that the introduction of 10% Cu_1Ni_1, Cu_1Ni_2, and Cu_1Ni_3 alloys lowers the onset temperature of H_2 desorption from MgH_2 to below 210 °C, and the H_2 desorption peak temperature is reduced to about 245 °C, which is more than 80 °C lower than the H_2 release temperature and peak temperature of ball-milled MgH_2, respectively. However, the dehydrogenation peak temperature of MgH_2 catalyzed by Cu_3Ni_1 rises to 266 °C with the Cu content in CuNi alloys exceeding 50%.

Comparing the TPD curves of MgH_2 catalyzed by CuNi and different amounts of Ni (Supplementary Figure 7c), with the same addition of 10%, the onset and peak temperatures of H_2 desorption from MgH_2 catalyzed by CuNi alloy decreased by more than 15 °C compared to the samples catalyzed by Ni. Moreover, MgH_2 catalyzed by 10% Cu_1Ni_1 exhibited a 35 °C lower onset temperature and peak temperature than that catalyzed by 5% Ni, indicating a significant catalytic improvement of CuNi on the dehydrogenation thermodynamics of MgH_2, which is not solely contributed by Ni in the catalyst. Even when the loading of Ni was increased to 20%, the dehydrogenation temperature did not further reduce, nor did it surpass the improvement achieved by CuNi. These results suggest that the introduction of Cu in CuNi alloys could further promote the catalytic improvement of Ni on decreasing the dehydrogenation temperature of MgH_2.

To further demonstrate the synergistic effect of CuNi alloys, equimolar Cu and Ni nanoparticles (Cu at 5.2% and Ni at 4.8%) are added separately into MgH_2 with a total catalyst loading remaining at 10 wt.%. The dehydrogenation peak temperature for equimolar Cu and Ni is 253.9 °C, which is 7.5 °C higher than that of Cu_1Ni_1 (246.6 °C). Additionally, the dehydrogenation onset temperature for equimolar Cu and Ni is 12.9 °C higher than that of Cu_1Ni_1 (196.9 °C), and the temperature range required for dehydrogenation is also broader. There results indicates that, compared to Cu_1Ni_1 alloys, equimolar ratios of Cu and Ni exhibit a weakened catalytic effect on MgH_2, which may be attributed to the lack of close contact between Cu and Ni, hindering the rapid formation of the catalytic phase $Mg_2Ni(Cu)$ ternary alloy during the dehydrogenation process.

The activation energy (E_a) for dehydrogenation of MgH_2 with different catalysts (selecting Cu_1Ni_1 and Cu_1Ni_3 as representatives of CuNi alloys due to the similar dehydrogenation performance) are quantitatively calculated (Supplementary Figure 9a) using the Kissinger equation by TPD curves under different heat rates (Supplementary Figure 8):

$$\ln(\beta/T_p^2) = A - E_a/RT_p \qquad \text{(Equation 1)}$$

where β and T_p are the heating rate and dehydrogenation peak temperature, respectively, A is a temperature-independent coefficient, and R is the universal gas constant.

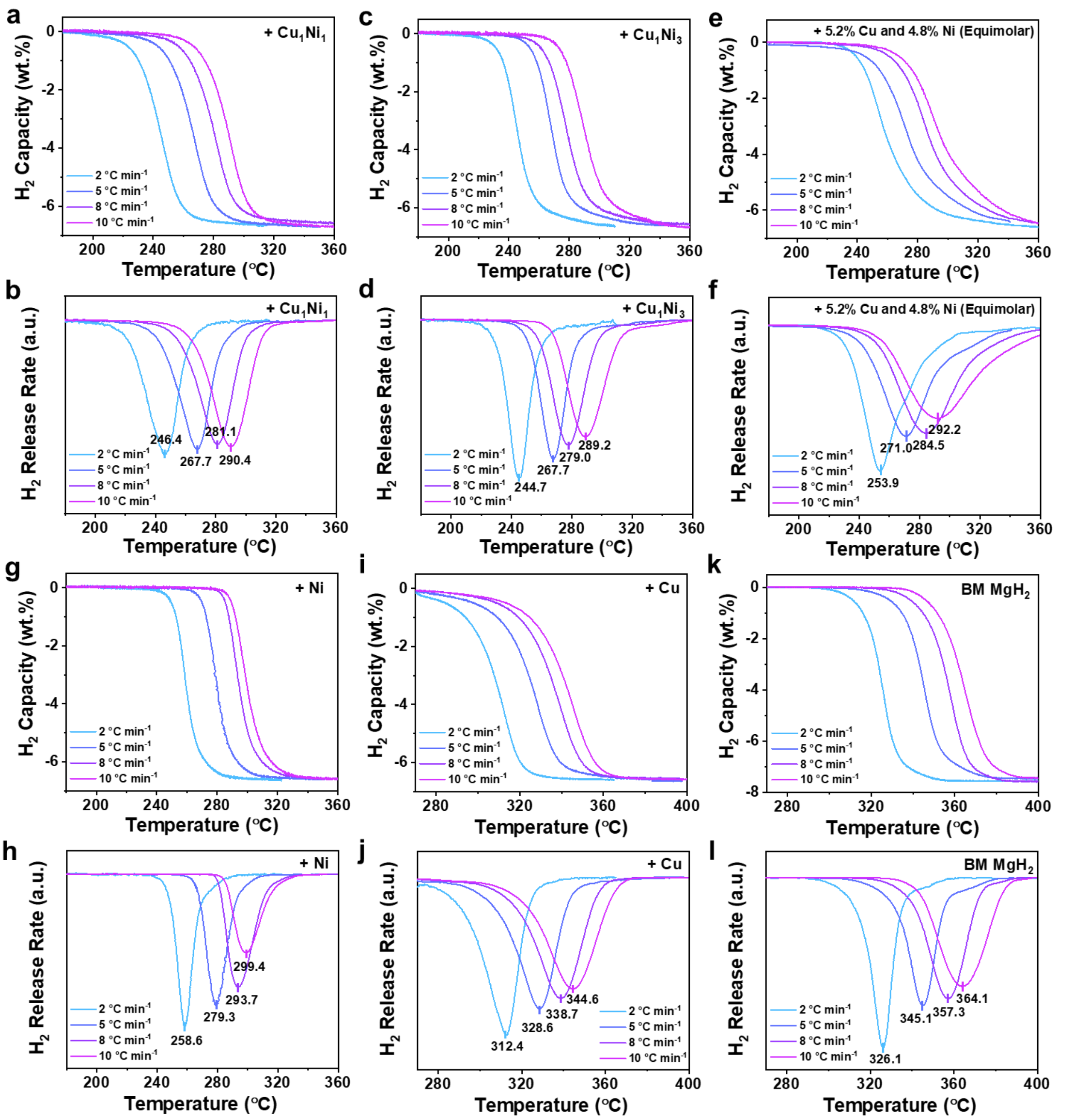

Supplementary Figure 8. TPD curves and their corresponding derivative curves. The curves of MgH_2 under the catalysis of a-b) Cu_1Ni_1, c-d) Cu_1Ni_3, e-f) equimolar Cu and Ni, g-h) Ni, i-j) Cu, including k-l) ball-milled MgH_2 for comparison. The unit a.u. means to arbitrary units. Source data are provided as a source data file.

a

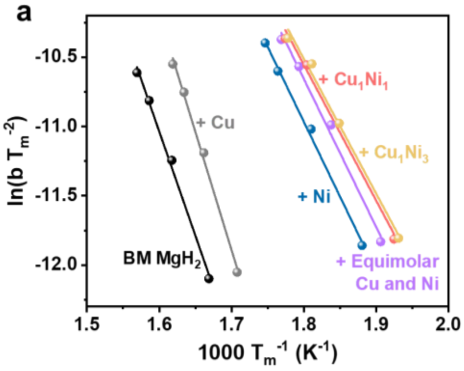

b

Samples	E_a (kJ mol^{-1} H_2)	R^2
Ball-milled MgH_2	125.04 ± 5.72	0.994
+10% Ni	89.79 ± 6.38	0.997
+10% Cu_1Ni_1	81.39 ± 5.18	0.992
+10% Cu_1Ni_3	80.81 ± 4.99	0.992
+10% Cu	141.67 ± 6.45	0.994
+ 5.2% Cu and 4.8% Ni (equimolar)	88.79 ± 5.37	0.992

Supplementary Figure 9. The comparison of heat-driven activation energies of dehydrogenation. a) Kissinger's plots and b) activation energies for dehydrogenation of MgH_2 under the catalysis of Cu_1Ni_1, Cu_1Ni_3, equimolar Cu and Ni, Ni, and Cu, including ball-milled MgH_2 for comparison. Source data are provided as a source data file.

The addition of Cu_1Ni_1 and Cu_1Ni_3 decreases the dehydrogenation activation energy of MgH_2 to 81.39 ± 5.18 kJ mol^{-1} and 80.81 ± 4.99 kJ mol^{-1}, respectively, which is not only much lower than that of pure MgH_2 without catalysts (125.04 ± 5.72 kJ mol^{-1}) but also lower than that of the Ni catalyzed sample (89.79 ± 6.38 kJ mol^{-1}). Moreover, the addition of equimolar Cu and Ni decreases the dehydrogenation activation energy of MgH_2 to 88.79 ± 5.37 kJ mol^{-1}, which is a little higher than that of the Cu_1Ni_1 catalyzed sample. Interestingly, the activation energy of Cu-catalyzed MgH_2 is even higher than that of pure MgH_2 without catalysts, indicating that pure Cu has no catalytic effect on MgH_2. These results further demonstrate the synergistic effect of Cu and Ni alloying on the improvement of MgH_2 dehydrogenation performance. And by comparison, MgH_2 catalyzed by CuNi alloys exhibits excellent performance in terms of both the temperature and activation energy of dehydrogenation, which are among the leading reported Ni-based or bimetallic-catalyst for MgH_2 hydrogen storage materials so far (Supplementary Table S3).

Supplementary Table 3. The performance of MgH_2 under catalysis as reported in some studies. Comparison of the onset and peak H_2 desorption temperature with the H_2 capacity of several MgH_2-catalyst hydrogen storage systems.

Dopants	Onset Temperature (°C)	Peak Temperature (°C)	E_a (kJ mol^{-1} H_2)	H_2 Capacity (wt.%)	Ref.
+ FeNi/rGO	230	273	93.6	6.9	S1
+ Ni_3Fe/BC	185	272	102	6.4	S2
+ $Fe_{0.64}Ni_{0.36}$@C	250	343	86.9	5.2	S3
+ Ni-Nb/rGO	198	230	57.9	6.4	S4
+ $NiMoO_4$	243	257	85.9	6.5	S5
+ NiV_2O_6	227	——	92.9	5.8	S6
+ $Ni_3(VO_4)_2$	210	280	72.6	5.5	S7
+ CoNi@C	173	254	82.3	6.2	S8
+ $NiTiO_3$	235	261	74	6.9	S9

+ PdNi metallene	200	263	62.5	6.4	S10
+ MoNi alloy	180	268	89.7	6.7	S11
+ NbTiC	195	230	80	6.8	S12
+ $TiNb_2O_7$	177	——	96	6.3	S13
+ Cu_1Ni_1 alloys	192.5	246.4	81.4	6.6	This work
+ Cu_1Ni_3 alloys	198.7	244.7	80.8	6.6	

Isothermal H_2 desorption kinetics curves at 250 °C (Fig. 2e, Supplementary Figure 7d) show that compared to MgH_2 without any dehydrogenation, MgH_2 with the addition of CuNi could achieve complete dehydrogenation at this temperature, indicating the improved dehydrogenation kinetics of MgH_2 by CuNi alloy. Among them, MgH_2 catalyzed by Cu_1Ni_3 releases a 6.3 wt.% H_2 in 20 minutes, while MgH_2 with Cu_1Ni_1 requires 40 minutes to reach the same H_2 release.

In terms of hydrogenation performance, the isothermal H_2 absorption kinetics curves at 250 °C showed that pure Mg could only achieve 77% of its theoretical hydrogen uptake, with a hydrogen absorption of 5.8 wt.% in 10 minutes under 1 MPa and 250 °C. However, the addition of Ni-based catalysts significantly improves the H_2 absorption kinetics of Mg. Mg catalyzed by Cu_1Ni_1, Cu_1Ni_2, and Cu_1Ni_3 absorb 6.1 wt.% H_2 within only 10 minutes after dehydrogenation, which is more than 95% of their maximum absorption capacity (Supplementary Figure 7e). It is worth noting that under the catalysis of Cu_1Ni_1, the sample could reversibly store 6.0 wt.% H_2 in Mg within 60 minutes at 150 °C (Supplementary Figure 7f), presenting the effective catalytic effect of CuNi alloys in improving the H_2 absorption kinetics of MgH_2. However, the hydrogenation rate is also relatively slower in the case of MgH_2 catalyzed by Cu_3Ni_1, leading to a deterioration in the catalytic effect, which is not conducive to achieving highly efficient solar-driven hydrogen storage. Therefore, Cu_3Ni_1 alloy is excluded and not further discussed in subsequent experiments.

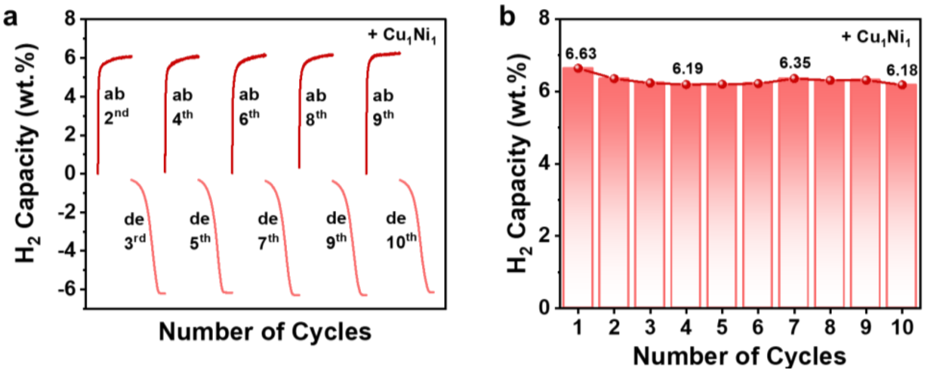

Supplementary Figure 10. The performance of heat-driven hydrogen storage cycling. a) Isothermal hydrogenation and dehydrogenation under 5 °C min^{-1} heat rate from 250 °C to 320 °C, and b) cycling H_2 capacity of MgH_2 under the catalysis of Cu_1Ni_1. The ab and de represent H_2 absorption and desorption, respectively. Source data are provided as a source data file.

In terms of cycling performance (Supplementary Figure 10a-b), after 10 cycles of H_2 absorption and desorption, MgH_2 catalyzed by Cu_1Ni_1 still retains 6.18 wt.% of reversible capacity, corresponding to a capacity retention of 96%, exhibiting its high-

capacity reversible stability.

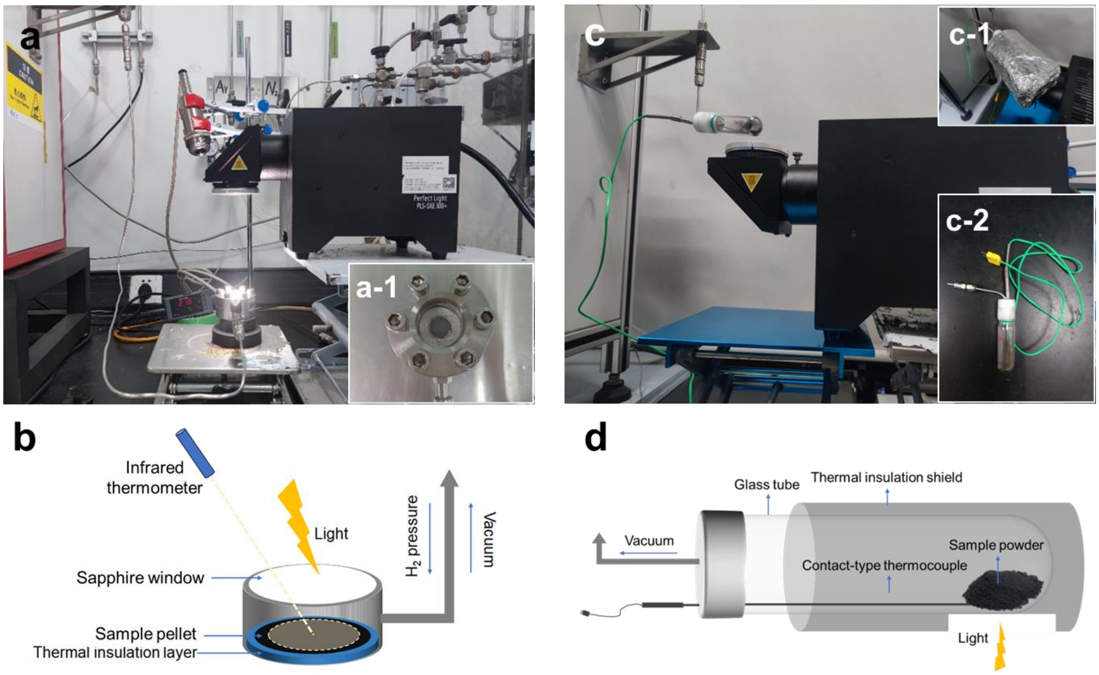

Supplementary Figure 11. Illustrations of two approaches for solar-driven measurement. The photograph of solar-driven H_2 absorption and desorption apparatus and schematic diagram of the customized reactor: a-b) high-pressure reactor with infrared thermometer used in this work, and c-d) glass-tube reactor with contact-type thermocouple used for temperature validation.

For temperature testing, the surface temperature during the H_2 absorption and desorption processes of the sample is continuously monitored in real-time *via* a short-wave infrared thermometer, which could receive the signal of infrared radiation from the sample's surface through the sapphire, and subsequently converts it into temperature data. Additionally, to minimize the influence of the unavoidable non-uniformity of photon flux of Xe lamp on temperature accuracy, we have adjusted the height of the infrared thermometer to nearly cover the entire sample range and ensure that the temperature measurement center aligns with the light source center, which provides a more accurate measurement of the sample surface’s average temperature.

Another detailed description for the hydrogen storage measurement and sample preparation of hydrogen-pressure reactor is provided in the "Hydrogen Storage Measurement" section of Method.

To compare the differences between the infrared thermometer and the contact-type thermocouple, a home-made glass tube reactor (Supplementary Figure 11c-d) that allows the direct insertion of a contact-type thermocouple is employed for hydrogen release tests without high pressure. Approximately 30 mg samples are placed in the reactor as powder to ensure that the thermocouple is fully covered by the materials to accurately measure the temperature of MgH_2 composite. During the testing process, a shield filled with thermal insulation cotton is used to minimize heat loss through thermal

conduction from glass tube to the outside (Supplementary Figure 11c-1).

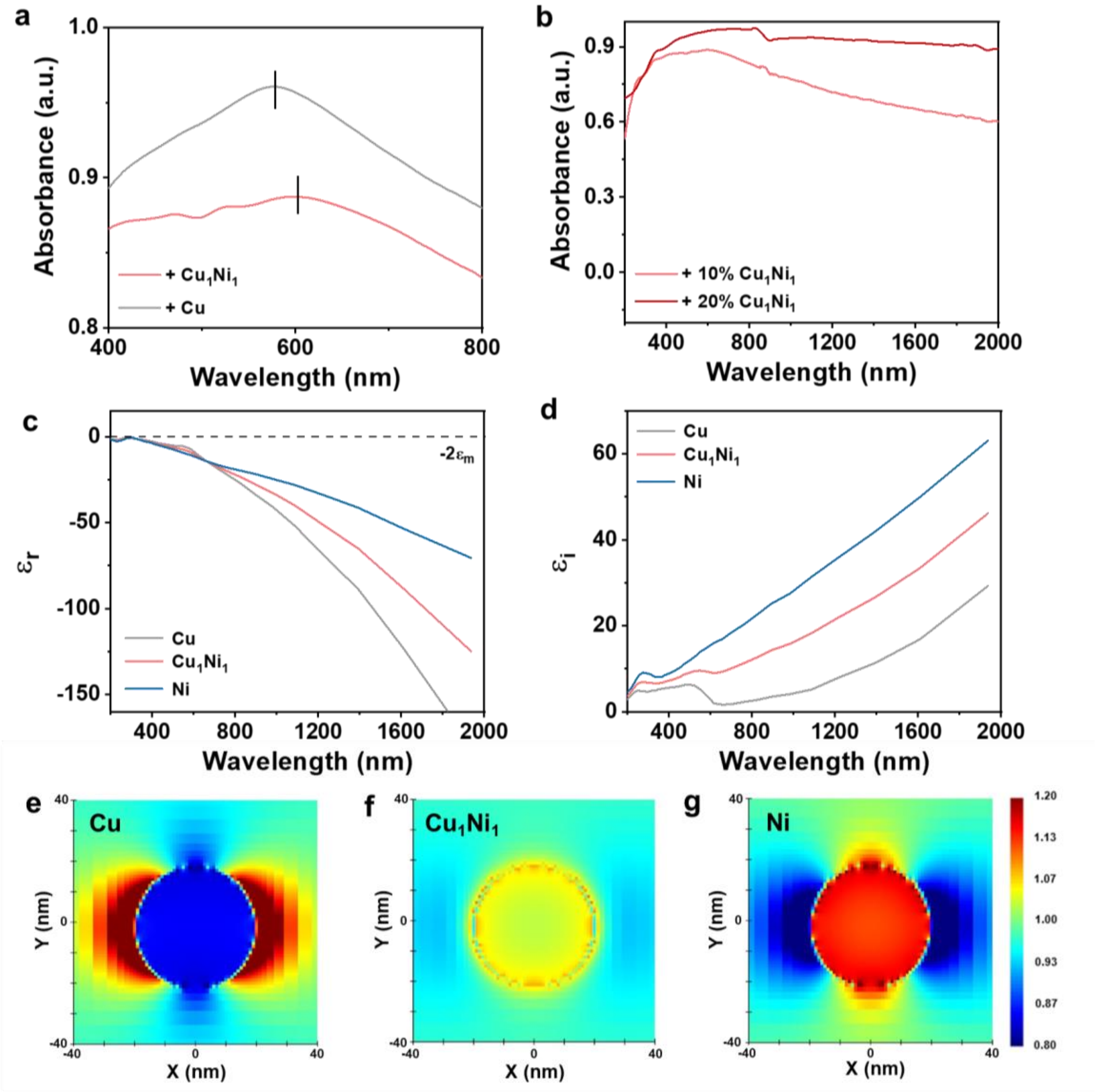

Supplementary Figure 12. FDTD simulation of CuNi alloy, Cu, and Ni. UV–*vis*–NIR absorption spectra of MgH_2 under the catalysis of a) Cu_1Ni_1 and Cu in the range of 400-800 nm and b) Cu_1Ni_1 with addition of 10% and 20%. The unit a.u. means to arbitrary units. c) Real and d) imaginary parts of dielectric functions of Cu, Cu_1Ni_1, and Ni metals. The dotted line represents the case where $\varepsilon_r \approx -2\varepsilon_m$, air. FDTD simulated localized electric field enhancement profiles of e) Cu， f) Cu_1Ni_1, and g) Ni. Source data are provided as a source data file.

The finite-difference time-domain (FDTD) simulation is performed by FDTD Package (Lumerical Solution, Inc.). Metal nanoparticles are set with a diameter of 20 nm. The simulation area is divided into uniform Yee cells with perfectly matched layer boundary condition [S14]. An incident plane wave is propagated from the top along *z* axis and polarized along the *x* axis, and a wavelength of 580 nm light is used for simulation. The process is performed at 1 nm mesh resolution within the period of 1000 fs.

The dielectric constants of Cu and Ni nanoparticles are assumed to be the same as that of the bulk metal and were obtained from Johnson and Christy [S15]. The dielectric constant (ε) of CuNi alloy is rarely available and hence, to describe the plasmonic properties, the dielectric constant of the alloys is calculated by the composition-weighted average method [S16, 17], where x_{Ni} is the Ni volume fraction, as given below:

$$\varepsilon_{\mathrm{alloy}}(\mathrm{x_{Ni}}, \omega) = \mathrm{x_{Ni}}\varepsilon_{\mathrm{Ni}}(\omega) + (1 - \mathrm{x_{Ni}})\varepsilon_{\mathrm{Cu}}(\omega) \qquad \text{(Equation 2)}$$

For Cu nanoparticles, plasmonic "hot spot" regions could be clearly observed around the particles (Supplementary Figure 12e), in contrast, Ni do not exhibit distinct "hot spot" regions near the periphery of the particles (Supplementary Figure 12g), which is related to the intrinsic electronic structure and optical properties of different metals [S18]. After the introduction of CuNi alloy, ring-shaped "hot spot" regions could be also observed near the periphery of alloy particles, but with an intensity weaker than that of Cu (Supplementary Figure 12f). FDTD results validate the strong LSPR-induced localized electric field of Cu and CuNi, confirming the characteristic absorption peaks of Cu and CuNi in the absorption spectrum.

Furthermore, many studies have also pointed out that the introduction of LSPR in metal particles leads to an overall increase in absorbance [S19, 20].This may be attributed to the fact that when the LSPR effect occurs, the free electrons in the metal nanoparticles undergo surface oscillations, causing a change in the electron density distribution near the surface. As a result, the effective optical cross-section of the metal particles increases, and metal nanoparticles could absorb more photons near the resonance frequency, thereby enhancing the overall absorbance [S21, 22]. This explains the enhanced overall absorption in the visible and NIR regions observed after the introduction of CuNi and Cu into the MgH_2 system, which increases with higher Cu content (Fig. 2b). When multi-metal nanoparticles are in close proximity, their LSPR effects couple with each other and form resonant states, leading to the scattering of the NIR by light interference with a more intense localized electric field and the increase of the overall absorbance [S23]. This is also evident in the improvement of absorption in the NIR region when increasing the catalyst content to 20% (Supplementary Figure 12b). Therefore, we believe that, after ball milling, the LSPR with the coupling effect of Cu and CuNi alloys are the primary factors for the overall enhancement of the absorbance of MgH_2 composites.

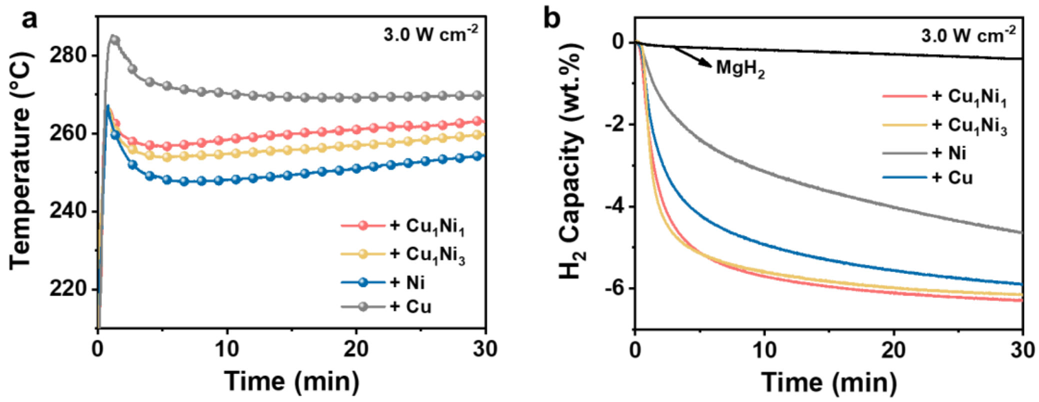

Supplementary Figure 13. Solar-driven dehydrogenation performance under high light intensity. a) The response of temperature to light irradiation and b) the corresponding H_2 desorption curves under 3.0 W cm^{-2} of MgH_2 under the catalysis of Cu_1Ni_1, Cu_1Ni_3, Ni, and Cu, including pure MgH_2 for comparison. Source data are provided as a source data file.

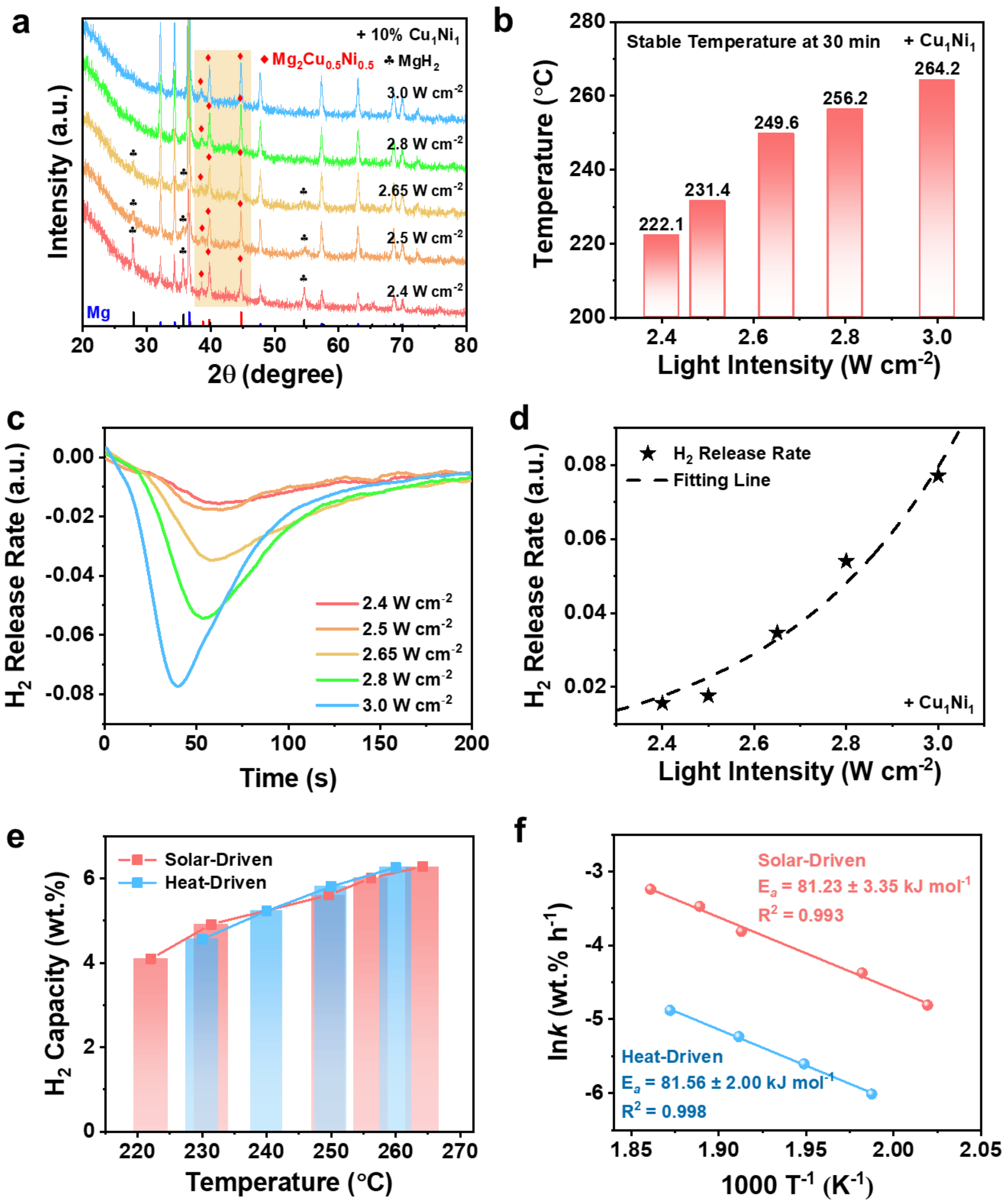

Supplementary Figure 14. Comparison of dehydrogenation using solar energy and thermal heating. a) XRD patterns after H_2 desorption, b) the surface temperature, c) the dehydrogenation instantaneous rate, and d) the fitting of H_2 release rate of MgH_2 under the catalysis of Cu_1Ni_1 using different light intensities. The unit a.u. means to arbitrary units. e) Comparison of the amount of H_2 desorption capacity and f) activation energies of MgH_2 under the catalysis of Cu_1Ni_1 using solar energy and thermal heating at different temperatures. Source data are provided as a source data file.

The activation energy of MgH_2 under the catalysis of Cu_1Ni_1 alloys under solar irradiation and heat is also fitted by the Johnson–Mehl–Avrami (JMA) equation joint with the Arrhenius equation. The JMA equation is expressed as:

$$\ln[-\ln(1-\alpha)] = \mathrm{n}\ln\mathrm{k} + \mathrm{n}\ln\mathrm{t} \quad \text{(Equation 3)}$$

where α is the fraction of Mg transformed into MgH_2 at reaction time t, and n and k represent the Avrami exponent and effective kinetic parameter, respectively. The

values of η and k can be deduced based on the isothermal kinetics data at different temperatures. And the E_a value could be calculated from the Arrhenius equation, written as:

$$\ln k = -E_a/RT + \ln A \qquad \text{(Equation 4)}$$

where T is the temperature of isothermal driven by heat or solar-driven hydrogen desorption and A is the pre-exponential factor.

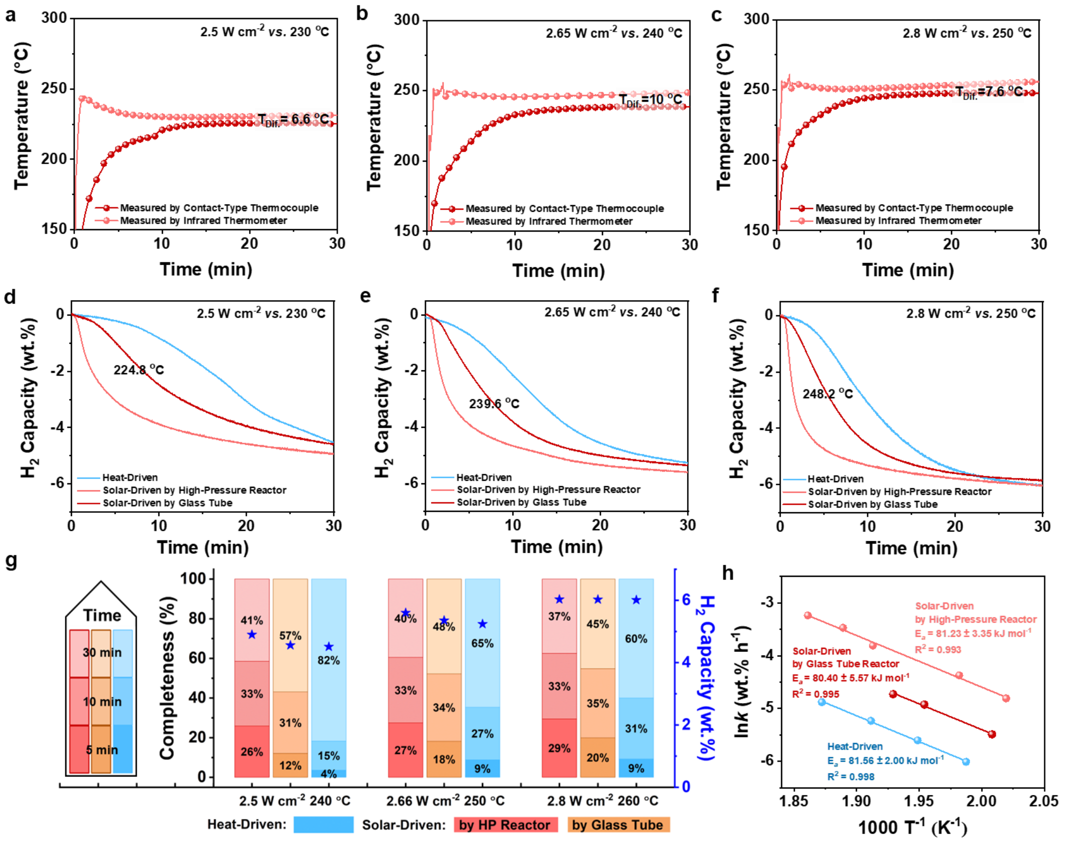

Supplementary Figure 15. Dehydrogenation performance of Cu_1Ni_1 samples driven by solar energy *via* two approaches with thermal heating for comparison. a-c) Temperature and d-f) corresponding dehydrogenation curves of MgH_2 catalyzed by Cu_1Ni_1 *via* two different measurement method under varying light intensity, in comparison of that driven by heating. g) Completeness ratio over time using solar energy *via* two different measurement method and direct heating with normalized H_2 release capacity of MgH_2 under the catalysis of Cu_1Ni_1 at varying light intensities (or corresponding temperatures). h) activation energies of MgH_2 under the catalysis of Cu_1Ni_1 using solar energy under different light intensities and thermal heating at different temperatures. Source data are provided as a source data file.

The difference of contact area (powder *vs.* pellet) with light between the samples, as well as the overall thermal management of the reactor, leads to the slower dehydrogenation rates of powder samples in the glass tube compared to the sample pellets in the high-pressure reactor. Additionally, powder splattering results in a portion of the powder not being maintained within a more uniformly illuminated range

(Supplementary Figure 11c-2), which also could contribute to the decrease in H_2 capacity.

The disparity of surface temperature between the two testing methods for all samples in our experiment falls within the range of 5~10 °C. The contact-type thermocouple takes approximately 10 minutes to reach a stable temperature, while the infrared thermometer responds more rapidly, which may contribute to the difference in measurement principle and sensitivity to some extent.

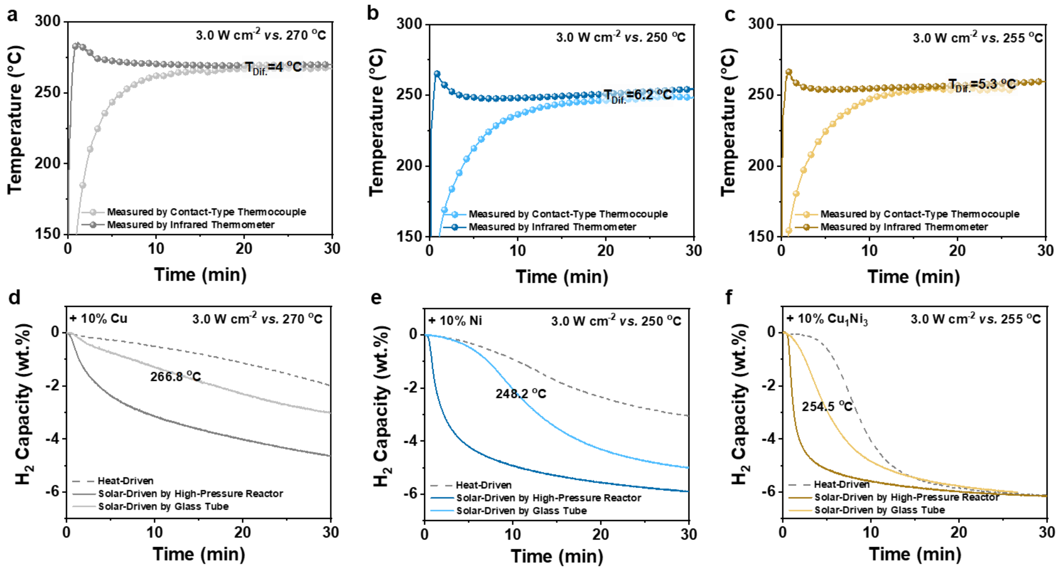

Supplementary Figure 16. Dehydrogenation performance of other samples driven by solar energy *via* two approaches with thermal heating for comparison. Temperature and corresponding dehydrogenation curves of MgH_2 catalyzed by a, e) Cu, b, d) Ni, and c, f) Cu_1Ni_3 *via* two different measurement method under varying light intensity, in comparison of that driven by heating. Source data are provided as a source data file.

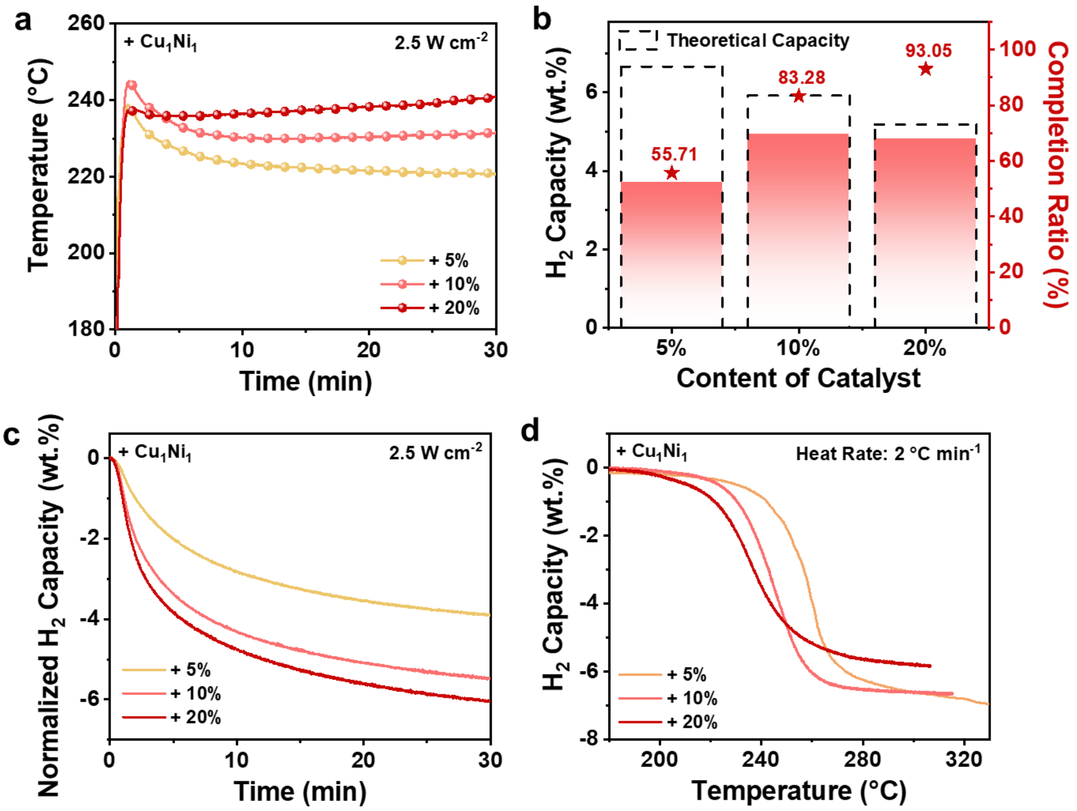

Supplementary Figure 17. Solar-driven dehydrogenation of MgH_2 under different Cu_1Ni_1 contents. a) Surface temperatures, b) the comparison of H_2 capacity with theoretical capacity and completion ratio, and c) the normalized H_2 desorption curves of MgH_2 under the catalysis of Cu_1Ni_1 with varying ratios using a light intensity of 2.5 W cm^{-2}. d) TPD curves of MgH_2 under the catalysis of Cu_1Ni_1 with varying ratios. Source data are provided as a source data file.

Nevertheless, the increase of the loading ratio of Cu_1Ni_1 would decrease the systematic H_2 storage capacity down to 4.82 wt.%, 0.11 wt.% lower than that of 10% Cu_1Ni_1. Therefore, the balance between the photothermal effect of CuNi alloys and the systematic H_2 storage capacity is of great significance to be optimized for practical applications, which also provides a basis for the selection of a loading ratio of 10% in this work.

Furthermore, MgH_2 catalyzed by 5% Cu_1Ni_1 reaches only a temperature of 221.7 °C with a dehydrogenation capacity of 3.71 wt.%, corresponding to a completion rate of merely 55.71%, similarly validating the indispensable role of *in-situ* generated $Mg_2Ni(Cu)$ in both photothermal and catalytic effects.

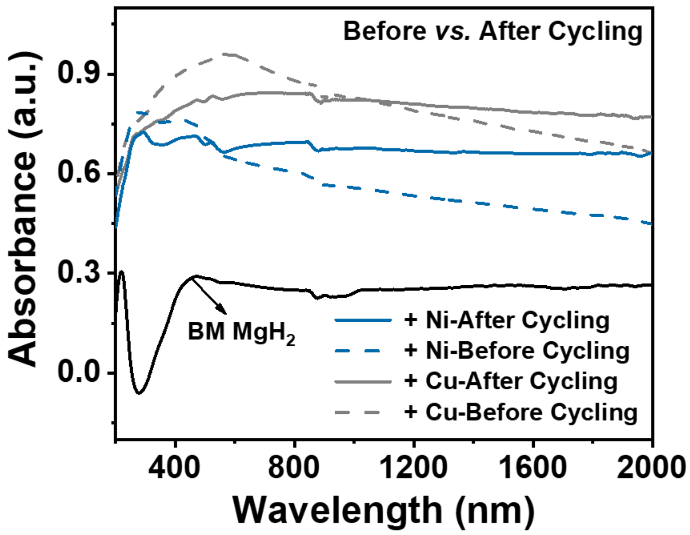

Supplementary Figure 18. Light absorption performance of single-metal samples before and after cycling. UV-*vis*-NIR absorption spectra of MgH_2 under the catalysis of Ni and Cu after cycling compared with its pristine ball-milled state (before cycling), including ball-milled MgH_2 for comparison. The unit a.u. means to arbitrary units. Source data are provided as a source data file.

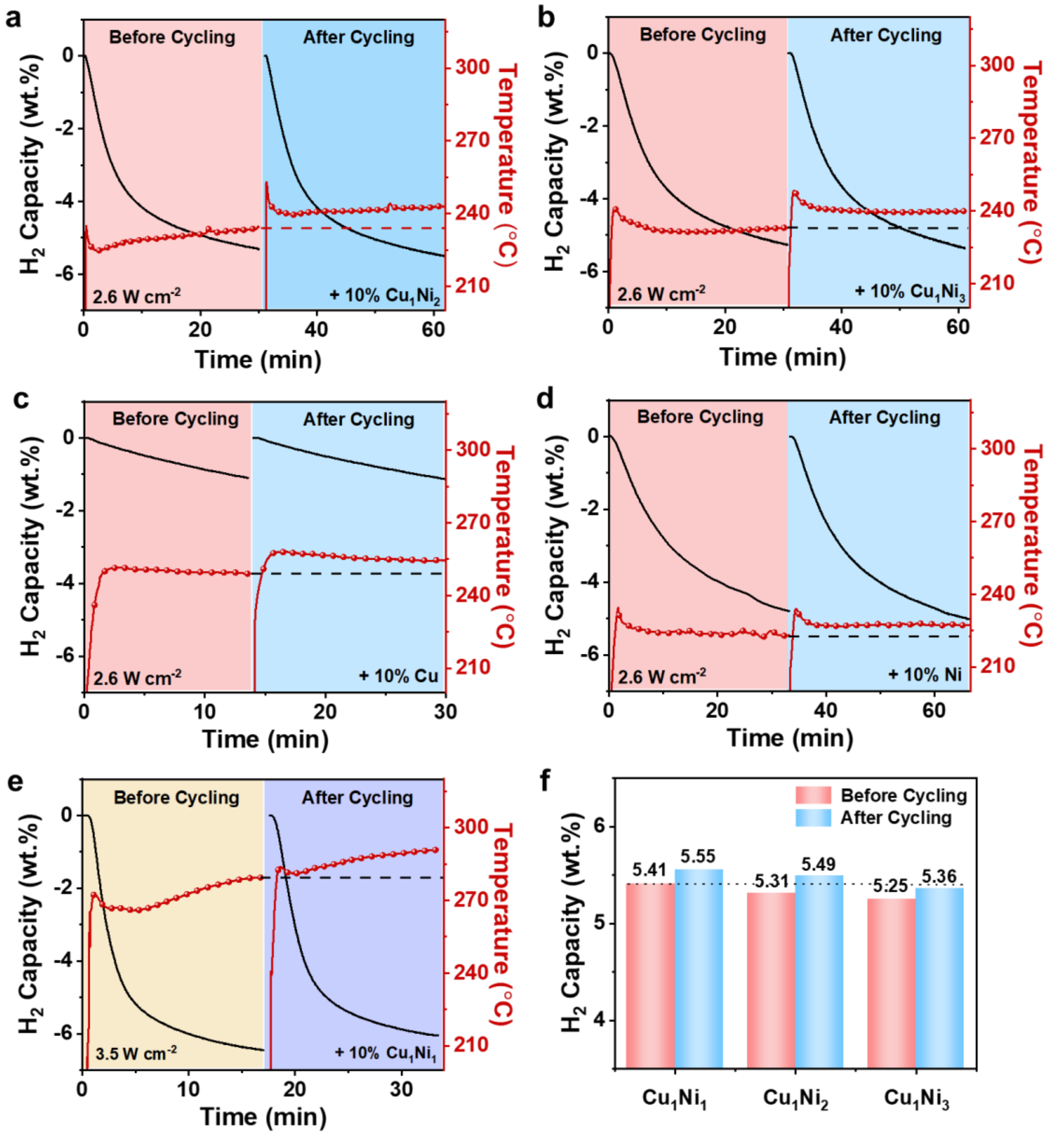

Supplementary Figure 19. The temperature and solar-driven dehydrogenation performance before and after cycling. The response of temperature with corresponding H_2 desorption curves for MgH_2 under the catalysis before and after cycling of a) Cu_1Ni_2, b) Cu_1Ni_3, c) Ni, and d) Cu under 2.6 W cm^{-2}, and e) Cu_1Ni_1 under 3.5 W cm^{-2}. f) The H_2 capacity to light irradiation of MgH_2 after cycling under the catalysis of Cu_1Ni_1, Cu_1Ni_2, Cu_1Ni_3, Ni, and Cu, respectively, with their pristine ball-milled state (before cycling) included for comparison. Source data are provided as a source data file.

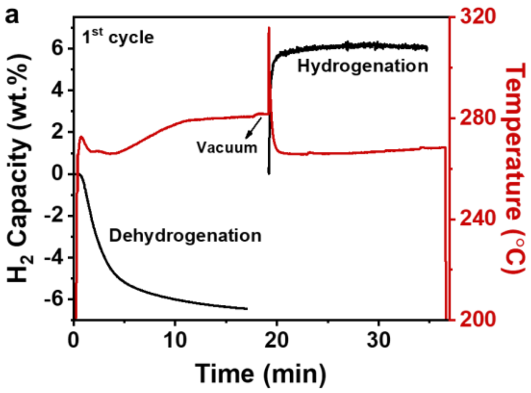

Supplementary Figure 20. The temperature and dehydrogenation curves for the first two cycles. a) The first and b) the second cycle of H_2 absorption and desorption curve of MgH_2 under the catalysis of Cu_1Ni_1 under solar irradiation. Source data are provided as a source data file.

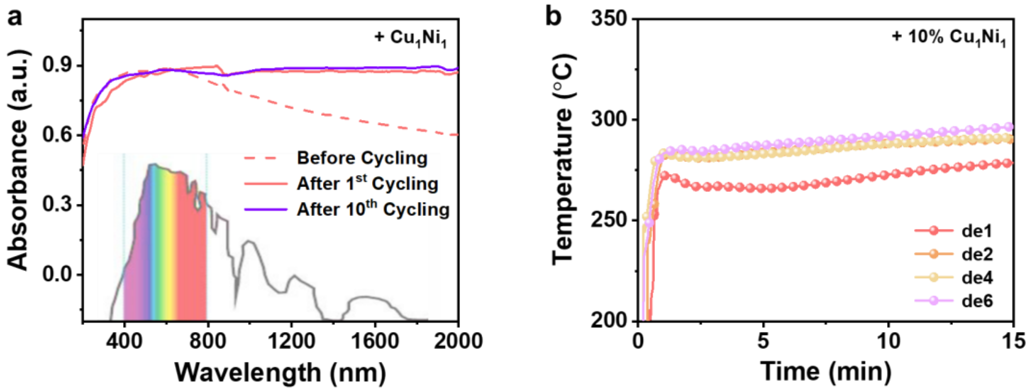

Supplementary Figure 21. The light absorption performance and temperature after cycling for different numbers of cycles. a) UV-*vis*-NIR absorption spectra of MgH_2 under the catalysis of Cu_1Ni_1 after 1st and 10th cycles of H_2 absorption. The unit a.u. means to arbitrary units. b) The surface temperature of MgH_2 under the catalysis of Cu_1Ni_1 at different times of dehydrogenation (de) during cycling. Source data are provided as a source data file.

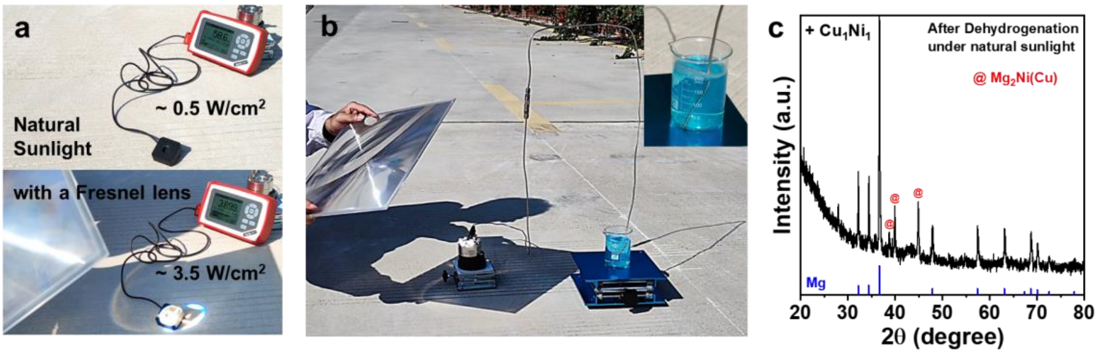

Supplementary Figure 22. Outdoor experiment by natural solar. a) Light intensity of natural solar without and with a commercial PMMA Fresnel lens. b) H_2 desorption experiment using natural solar as the energy source. The inset image shows the enlarged hydrogen bubbles. c) XRD patterns of the sample pellet after H_2 desorption under natural light. The unit a.u. means to arbitrary units. Source data are provided as a source data file.

The PMMA Fresnel lens measures 34.5 cm × 34.5 cm, with a thickness of 5 mm and a focal length of 35 cm, which costs around 14 US dollars. The surface has ring-shaped patterns from cold pressing, with a 93% light transmittance.

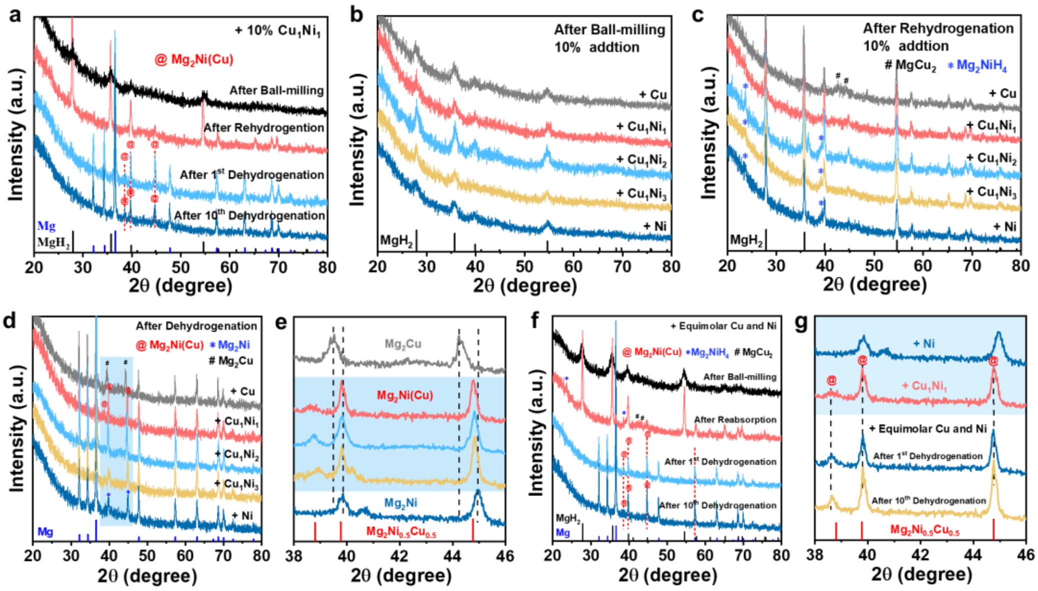

Supplementary Figure 23. Phase characterization of different samples and under different conditions. a) XRD patterns of MgH_2 under the catalysis of 10% Cu_1Ni_1 at different states. XRD patterns of MgH_2 b) after ball-milling, c) after rehydrogenation, and after dehydrogenation under the catalysis of Cu_1Ni_1, Cu_1Ni_2, Cu_1Ni_3, Ni, and Cu, in the 2θ range of d) 20~80 degrees and e) an enlarged view of 38~46 degrees. XRD patterns of MgH_2 after dehydrogenation under the catalysis of equimolar Cu and Ni, in the 2θ range of f) 20~80 degrees and g) an enlarged view of 38~46 degrees, including Cu_1Ni_1 and Ni for comparison. The unit a.u. means to arbitrary units. Source data are provided as a source data file.

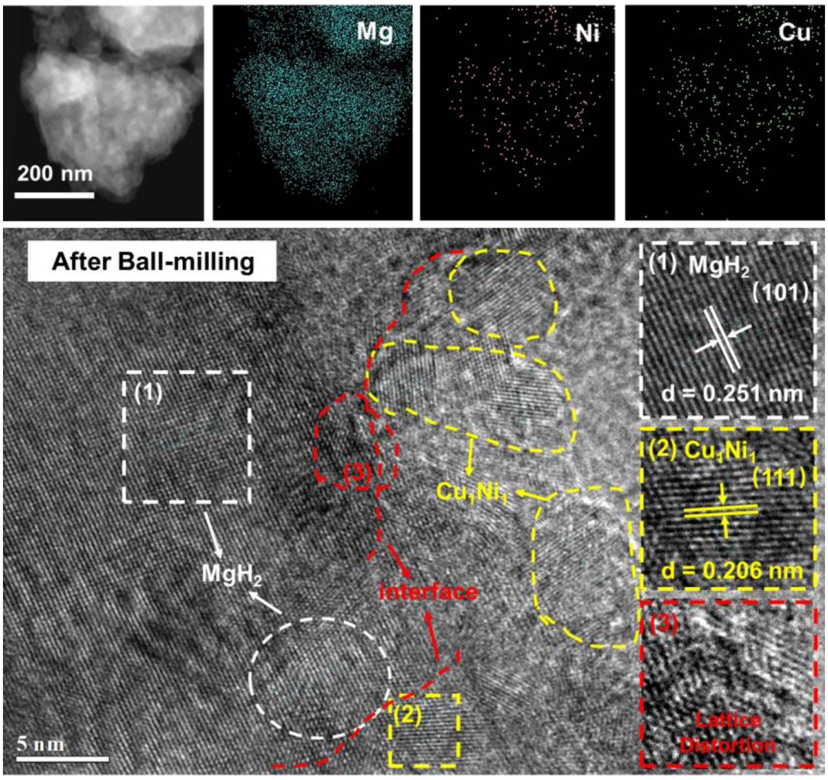

Supplementary Figure 24. Local microstructural characterization after ball-milling. TEM image of MgH_2 under the catalysis of Cu_1Ni_1.

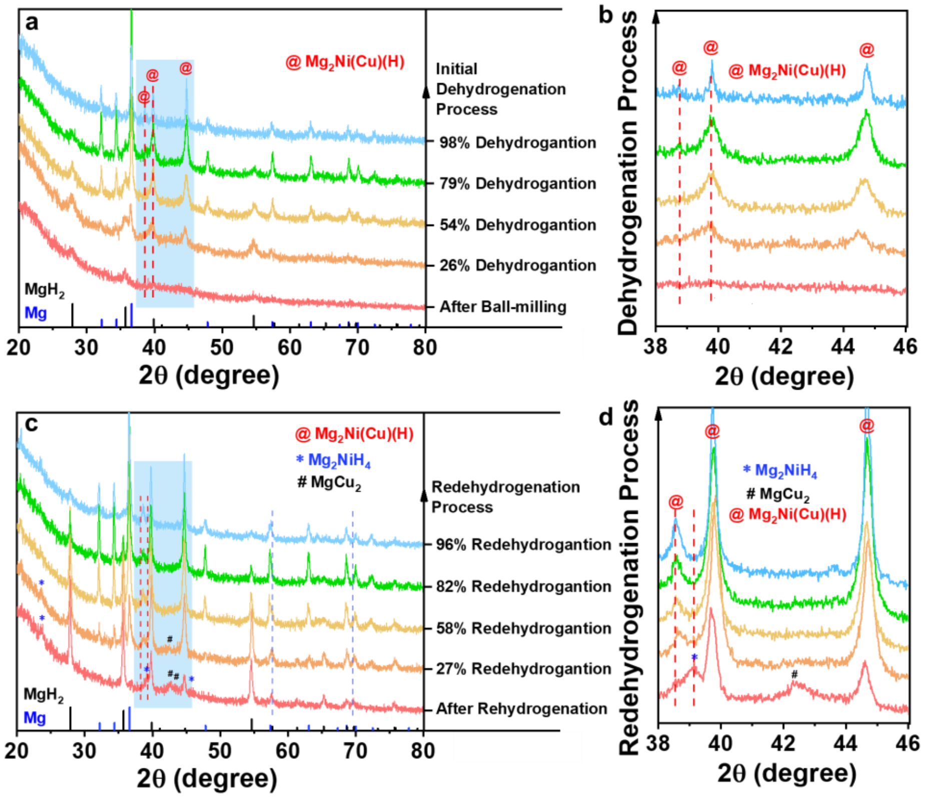

Supplementary Figure 25. Phase characterization during reaction process of Cu_1Ni_1 samples. XRD patterns of MgH_2 under the catalysis of Cu_1Ni_1, a-b) during initial dehydrogenation process and c-d) during re-dehydrogenation process in the 2θ range of 20~80 degrees and an enlarged view of 38~46 degrees. Source data are provided as a source data file.

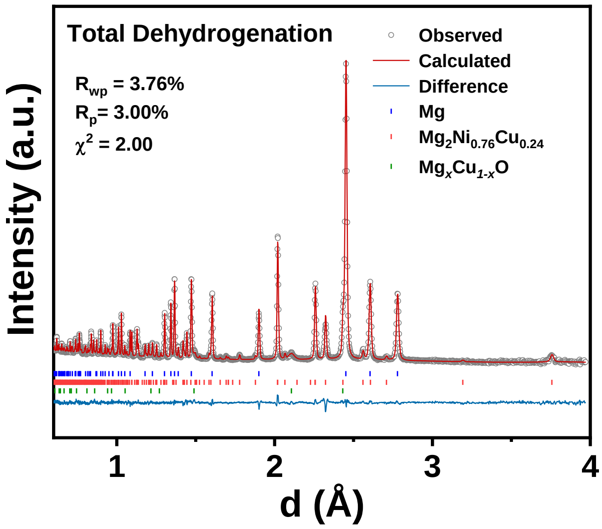

Supplementary Figure 26. Powder neutron diffraction and Rietveld refinement profiles. The results of total dehydrogenation states of MgH_2 under the catalysis of CuNi alloys. The unit a.u. means to arbitrary units. Source data are provided as a source data file.

Supplementary Table 4. The phase composition fractions and space groups. The results of total dehydrogenation of MgH_2 under the catalysis of CuNi alloy.

	Space Group	Total Dehydrogenation
Mg	$P6_3/mmc$	64.58%
$Mg_2Ni_{1-x}(Cu)_x$	$P6_222$	32.00%
MgD_2	$P4_2/mnm$	0.00%
$Mg_xCu_{1-x}O$	Fm-3m	3.42%

Supplementary Table 5. Rietveld refinement parameters. Summary of parameters in the $P6_222$ Cell for $Mg_2Ni_{1-x}Cu_x$ obtained from powder neutron diffraction.

**Atomic positions for $Mg_2Ni_{0.76}Cu_{0.24}$ of total dehydrogenation state** (a = 5.214 Å, b = 5.214 Å, c = 13.540 Å, α = β = 90°, γ = 120°)						
	**Wyckoff Position**	***x***	***y***	***z***	**Occupancy**	**U_{iso}**
**Mg1**	6f	0.500	0.000	0.386	1.000	0.01014
**Mg2**	6j	0.158	0.316	0.500	1.000	0.01014
**Ni1**	3c	0.500	0.000	0.000	0.608	0.00898
**Ni2**	3a	0.000	0.000	0.000	0.914	0.01910
**Cu1**	3a	0.000	0.000	0.000	0.861	0.01910
**Cu2**	3c	0.500	0.000	0.000	0.3920	0.00898

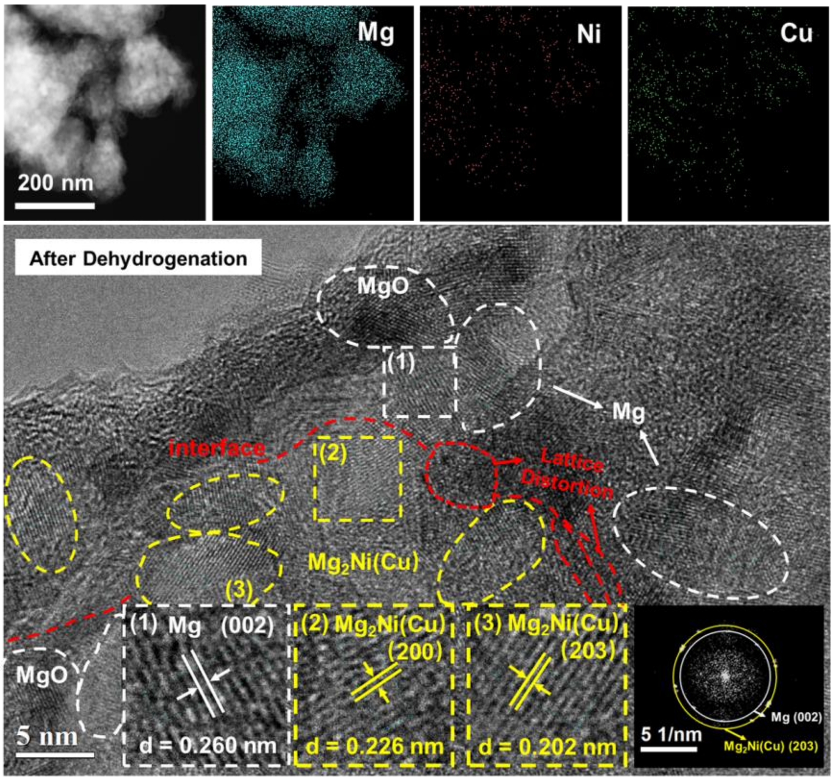

Supplementary Figure 27. Local microstructural characterization after dehydrogenation. TEM image of MgH_2 under the catalysis of Cu_1Ni_1.

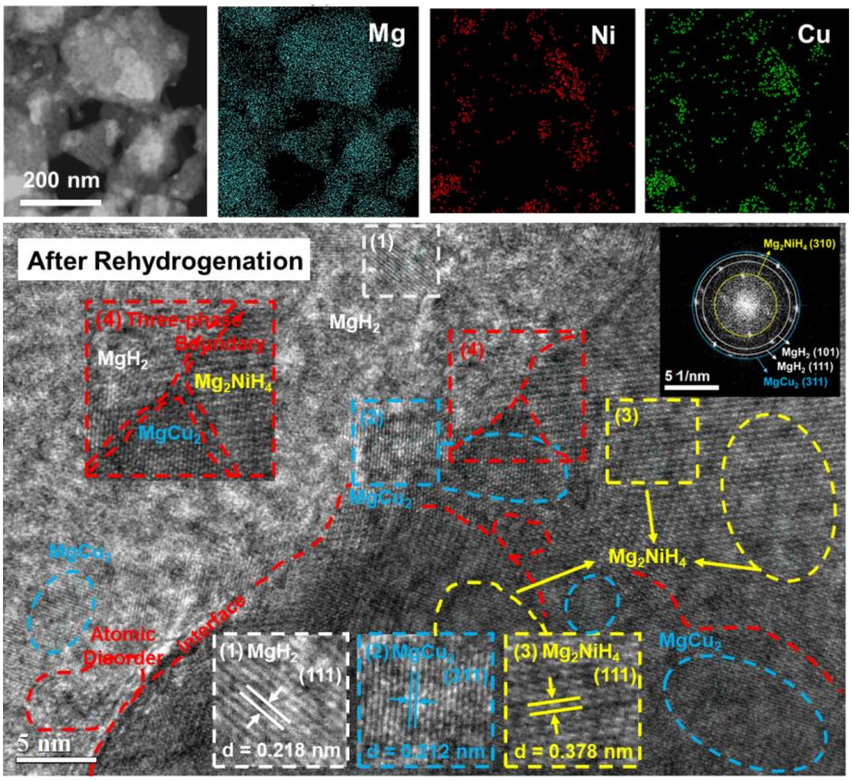

Supplementary Figure 28. Local microstructural characterization after rehydrogenation. TEM image of MgH_2 under the catalysis of Cu_1Ni_1.

Supplementary Table 6. Theoretical H_2 capacity after reaction with different content of Cu. The theoretical rehydrogenation with H_2 capacity maintenance rate of Mg under the catalysis of CuNi alloys, Cu, and Ni, including pure MgH_2 for comparison.

Samples	Theoretical H_2 Absorption of Mg in System	Theoretical H_2 Capacity Maintenance Rate
MgH_2	1 Mg: 1 H_2	100.0%
+10% Ni	1 Mg: 1 H_2	100.0%
+10% Cu_1Ni_1	90% (1 Mg: 1 H_2) + 10% (1 Mg: 0.875 H_2)	98.8%
+10% Cu_1Ni_2	90% (1 Mg: 1 H_2) + 10% (1 Mg: 0.917 H_2)	99.2%
+10% Cu_1Ni_3	90% (1 Mg: 1 H_2) + 10% (1 Mg: 0.938 H_2)	99.4%
+10% Cu	90% (1 Mg: 1 H_2) + 10% (1 Mg: 0.750 H_2)	97.5%

The addition of Cu is detrimental to the retention of the system's theoretical H_2 storage capacity of MgH_2, with 97.5% H_2 maintenance rate under 10% Cu catalyst. While Mg_2NiH_4 is formed for MgH_2 under the catalysts of Ni, which possesses the same theoretical H_2 absorption capacity per Mg unit as pure MgH_2, and thus it does not result in a significant loss of the system's H_2 capacity. As for CuNi alloys, the theoretical H_2 capacity maintenance rate of MgH_2 under the catalysts of Cu_1Ni_1, Cu_1Ni_2, and Cu_1Ni_3 is 98.8%, 99.2%, and 99.4%, respectively. Therefore, a loss in H_2 capacity of the system could nearly be neglected under the catalysts of CuNi alloys with low loading ratios (≤ 10%).

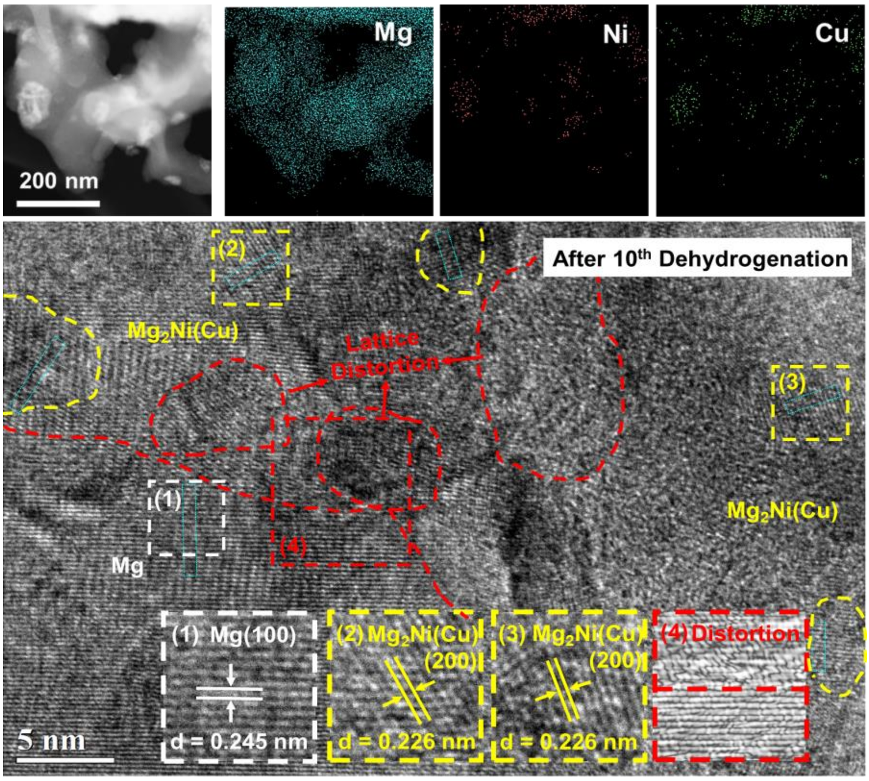

Supplementary Figure 29. Local microstructural characterization after 10th cycling. TEM image of MgH_2 under the catalysis of Cu_1Ni_1 after 10th H_2 desorption.

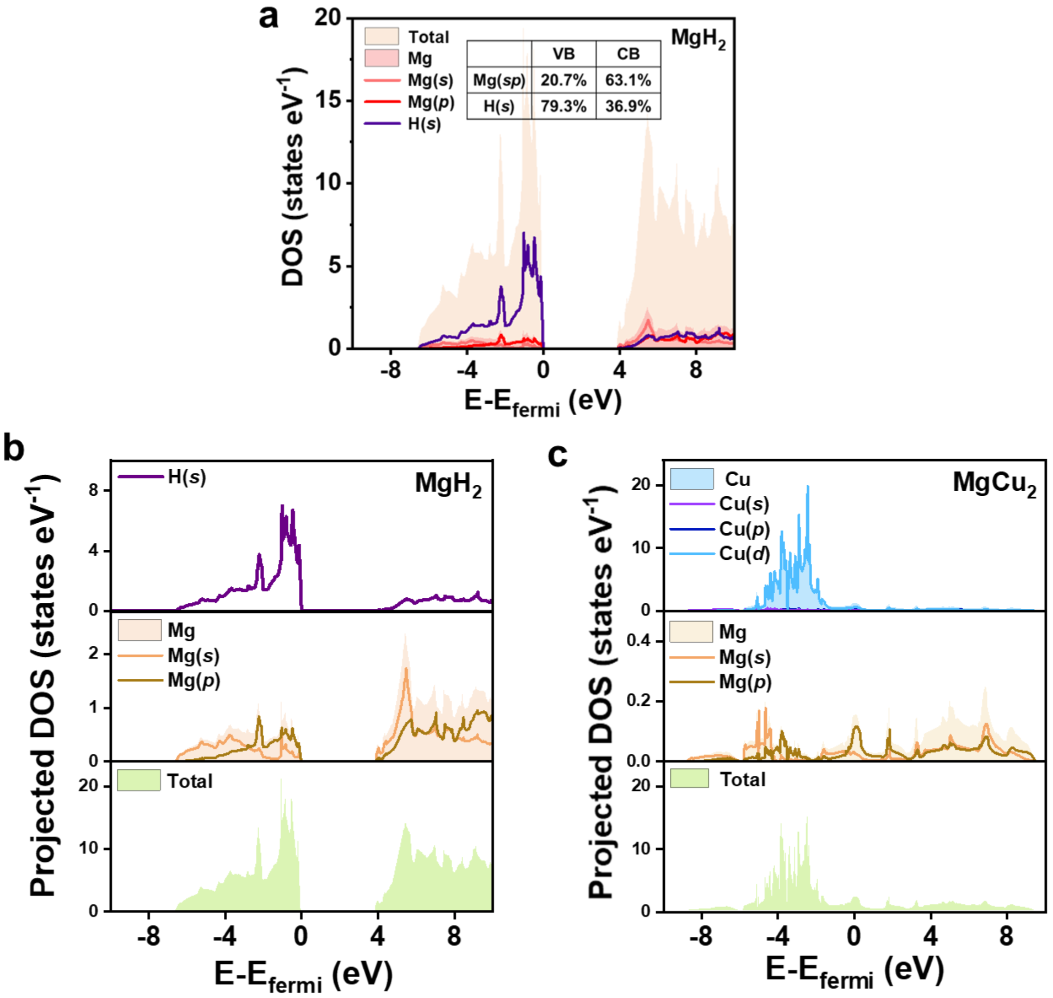

Supplementary Figure 30. Partial densities of states of MgH_2 and $MgCu_2$. a) Densities of states of Mg and H orbitals for MgH_2 with the calculated contribution percentages at the valence band and the conduction band. Partial densities of states of corresponding atoms for b) MgH_2 and c) $MgCu_2$. Source data are provided as a source data file.

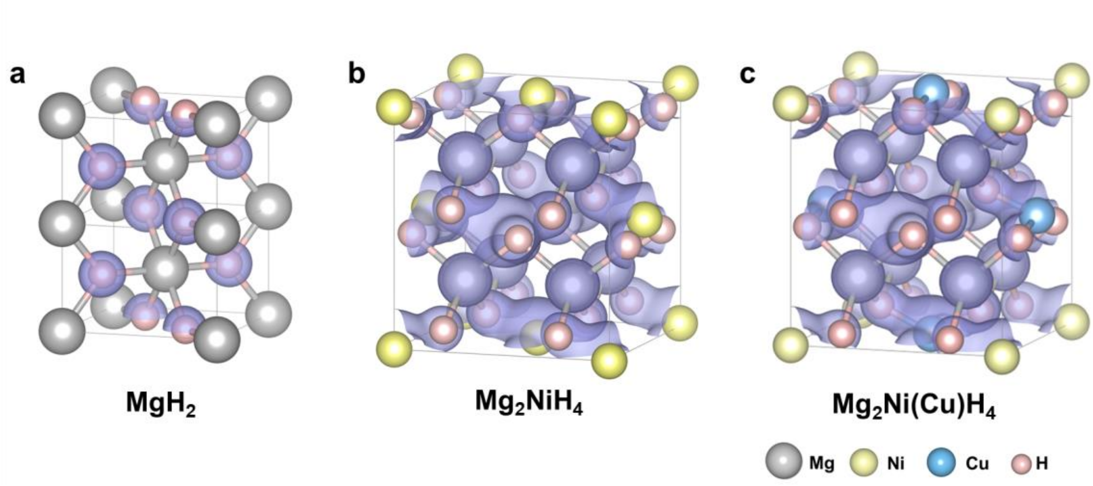

Supplementary Figure 31. Calculated charge densities of three hydrides. Isosurfaces of the calculated charge densities with an isovalue of 0.05 e Å^{-3} for a) MgH_2, b) Mg_2NiH_4 and c) $Mg_2Ni(Cu)H_4$.

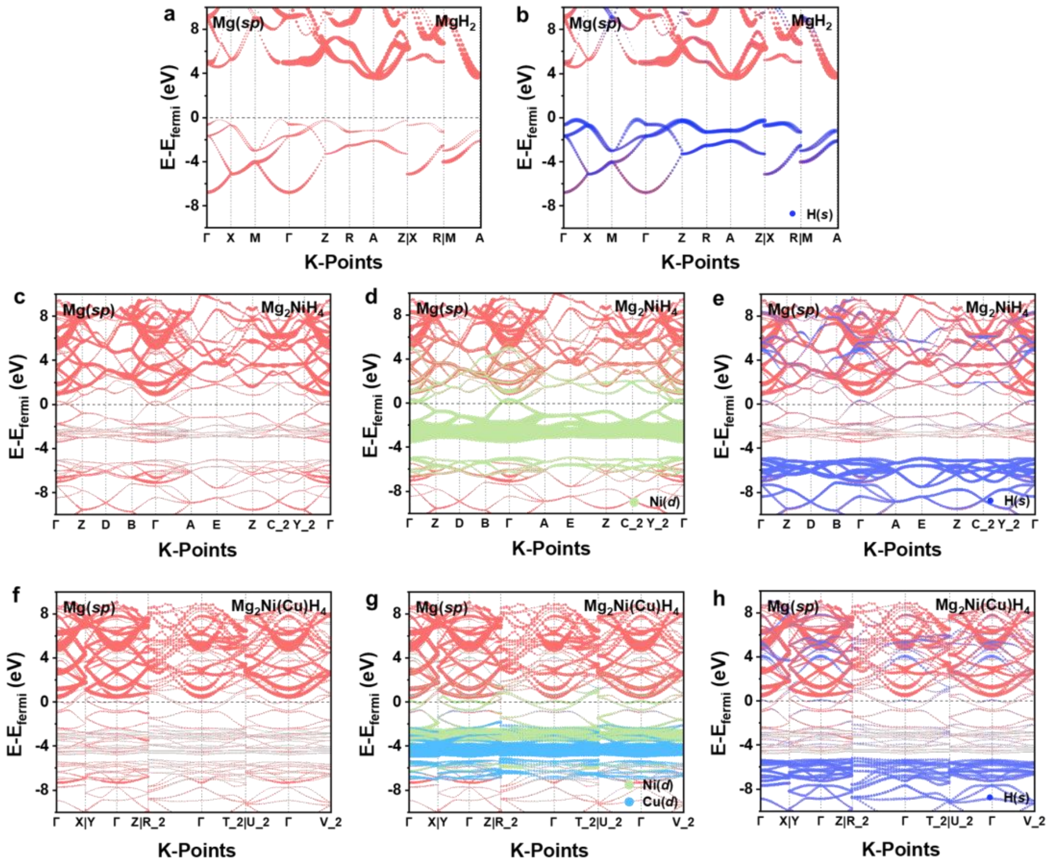

Supplementary Figure 32. The impact of other atoms on the distribution of Mg(*sp*) orbitals. Band structures of Mg(*sp*)-orbitals of MgH_2 a) without other orbitals and b) with H(*s*). Band structures of Mg(*sp*)-orbitals of Mg_2NiH_4 c) without other orbitals, d) with Ni(*d*), and e) H(*s*). Band structures of Mg(*sp*)-orbitals of $Mg_2Ni(Cu)H_4$ f) without other orbitals, g) with Ni(*d*) and Cu(*d*), and h) H(*s*). Source data are provided as a source data file.

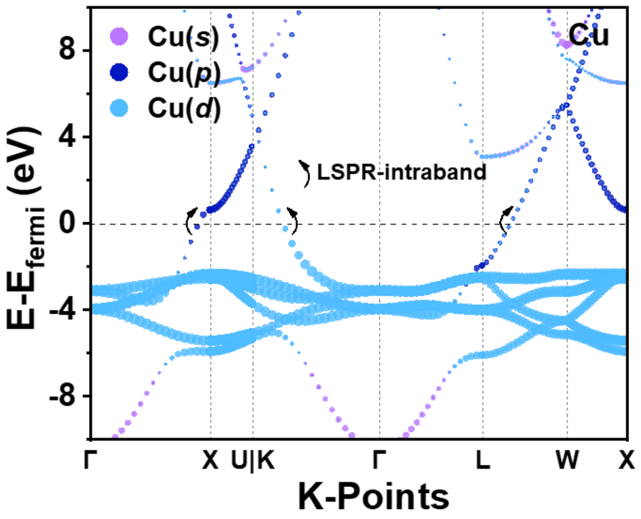

Supplementary Figure 33. Band structure of Cu nanoparticles with orbitals of *s*, *p*, and *d*. Source data are provided as a source data file.

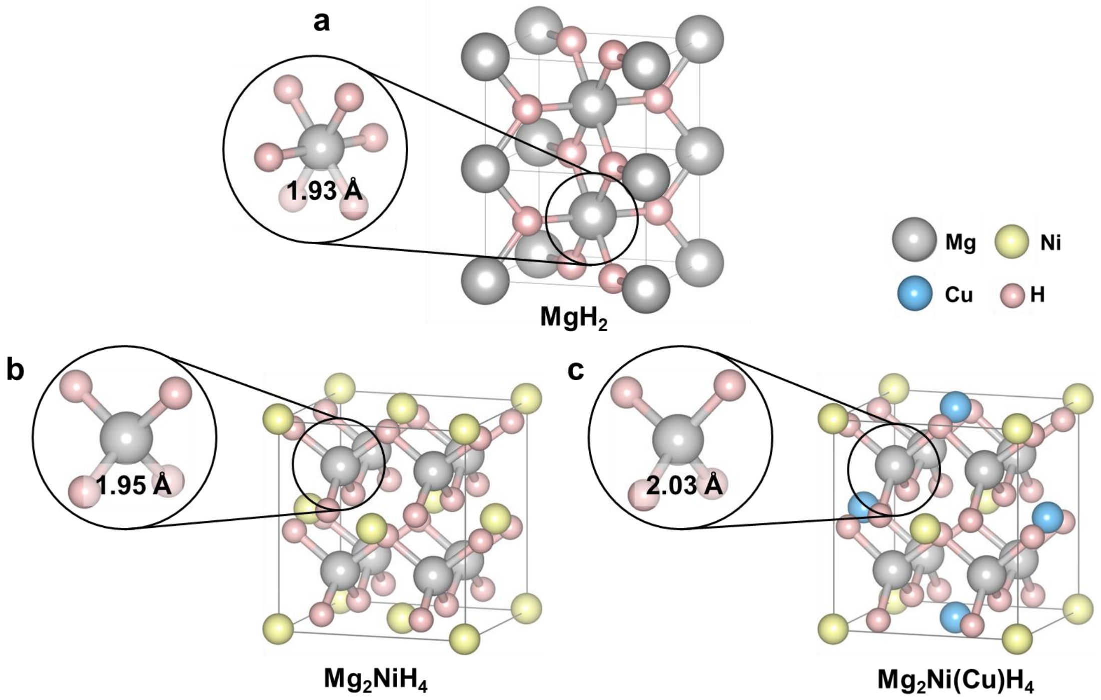

Supplementary Figure 34. The Mg-H bond length of the hydride phase after atomic doping. The length of Mg-H bond in a) MgH_2, b) Mg_2NiH_4 and c) $Mg_2Ni(Cu)H_4$.

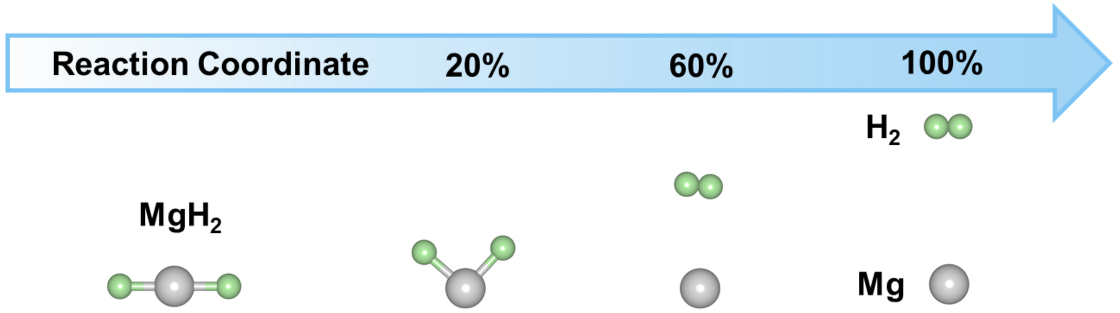

Supplementary Figure 35. Schematic illustration of self-dehydrogenation pathway of MgH_2.

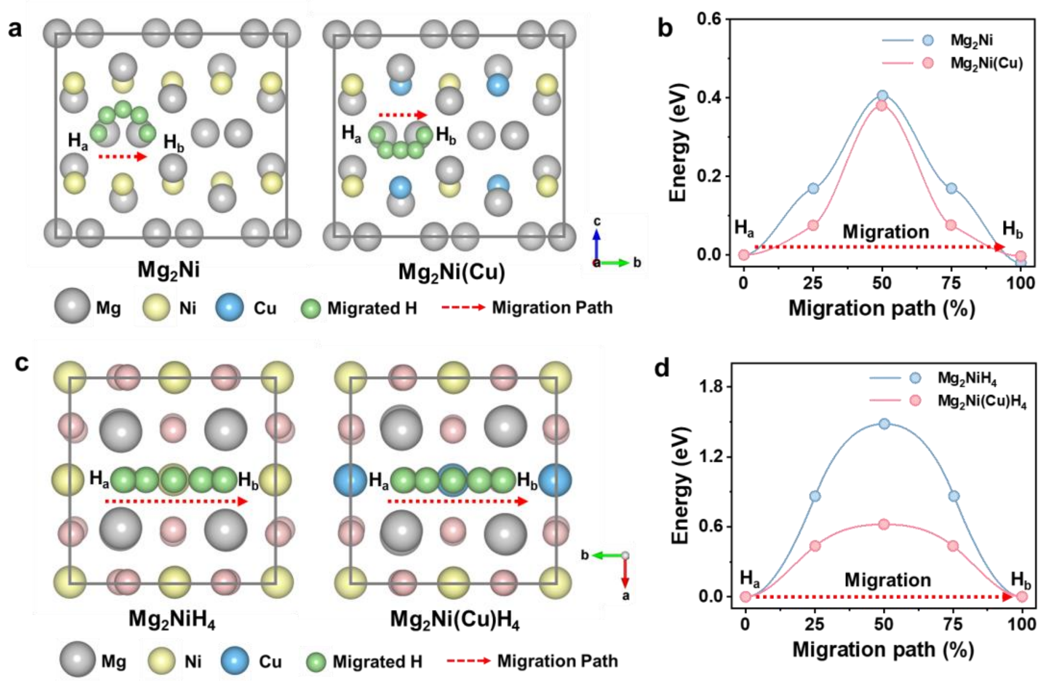

Supplementary Figure 36. H diffusion model with energy profiles. a) H diffusion model in Mg_2Ni and $Mg_2Ni(Cu)$ and b) corresponding energy profiles. c) H diffusion model in Mg_2NiH_4 and $Mg_2Ni(Cu)H_4$ and d) corresponding energy profiles. Source data are provided as a source data file.

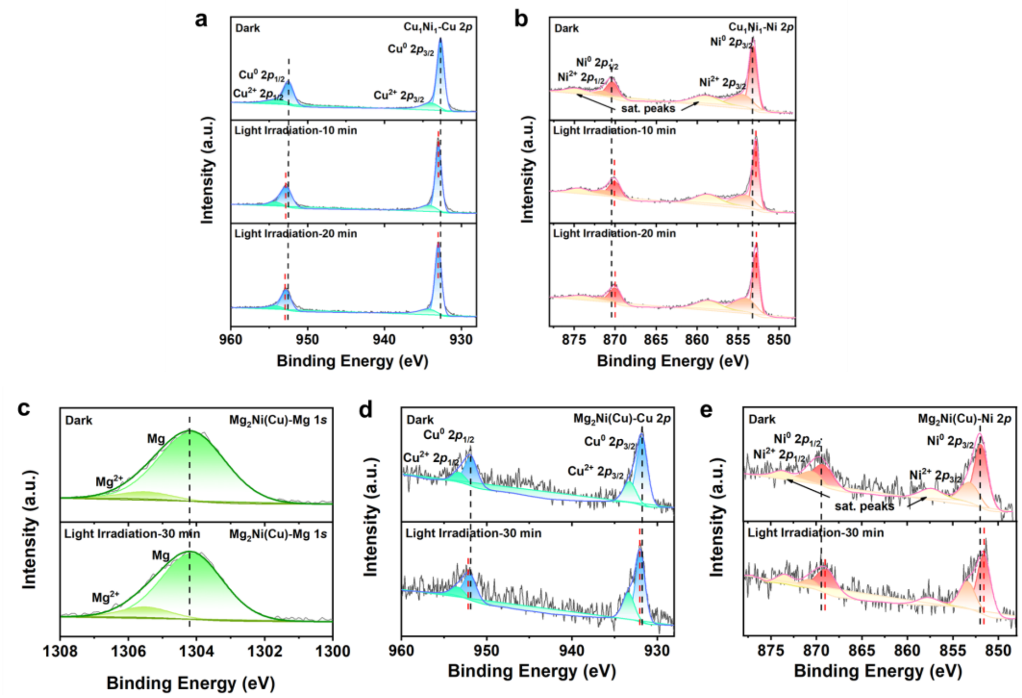

Supplementary Figure 37. XPS spectra of CuNi and $Mg_2Ni(Cu)$ alloy with and without solar irradiation. High-resolution a) Cu *2p* and b) Ni *2p* XPS spectra of Cu_1Ni_1 in the dark and under solar irradiation of 10 and 20 minutes. High-resolution c) Mg *1s,* d) Cu 2*p*, and e) Ni *2p* XPS spectra of $Mg_2Ni(Cu)$, the dehydrogenation state of MgH_2 catalyzed by 30% Cu_1Ni_1 alloy, in the dark and under solar irradiation. The unit a.u. means to arbitrary units. Source data are provided as a source data file.

After the *in-situ* formation of $Mg_2Ni(Cu)$ alloy during the initial dehydrogenation, the relative transformation of hot electrons between Cu and Ni persists under light irradiation. However, no observable changes in the binding energy of Mg atoms are observed, attributed to the lower work function of Mg (3.68 eV) compared to Cu (Supplementary Figure 37c-e).

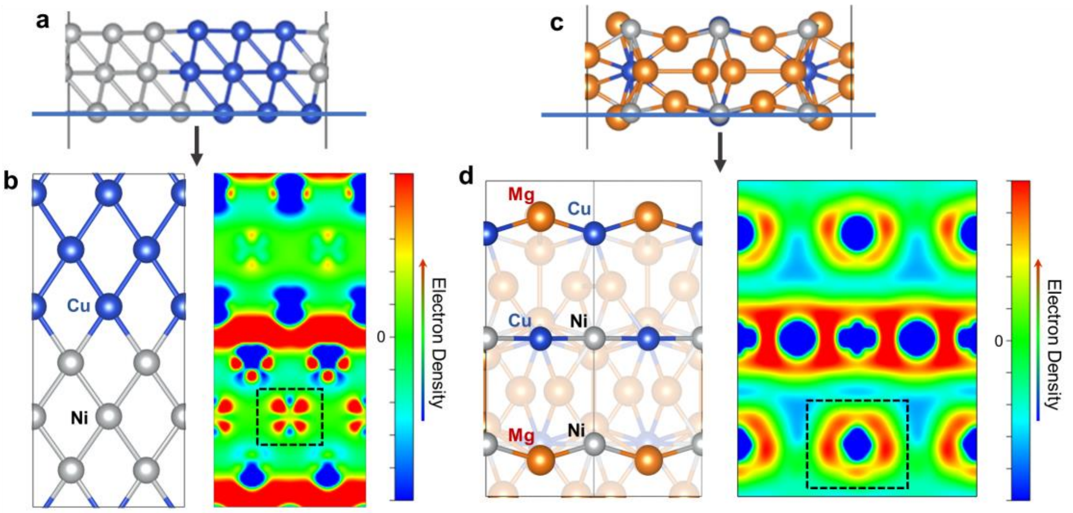

Supplementary Figure 38. Simulation of electron distribution around Ni atoms. Crystal model and selected plane of a) CuNi and c) $Mg_2Ni(Cu)$ alloy. Distribution of atoms and corresponding differential charge densities of b) CuNi alloy and d) $Mg_2Ni(Cu)$ alloy. All the results are plotted with an isovalue of 0.001 e $Bohr^{-3}$.

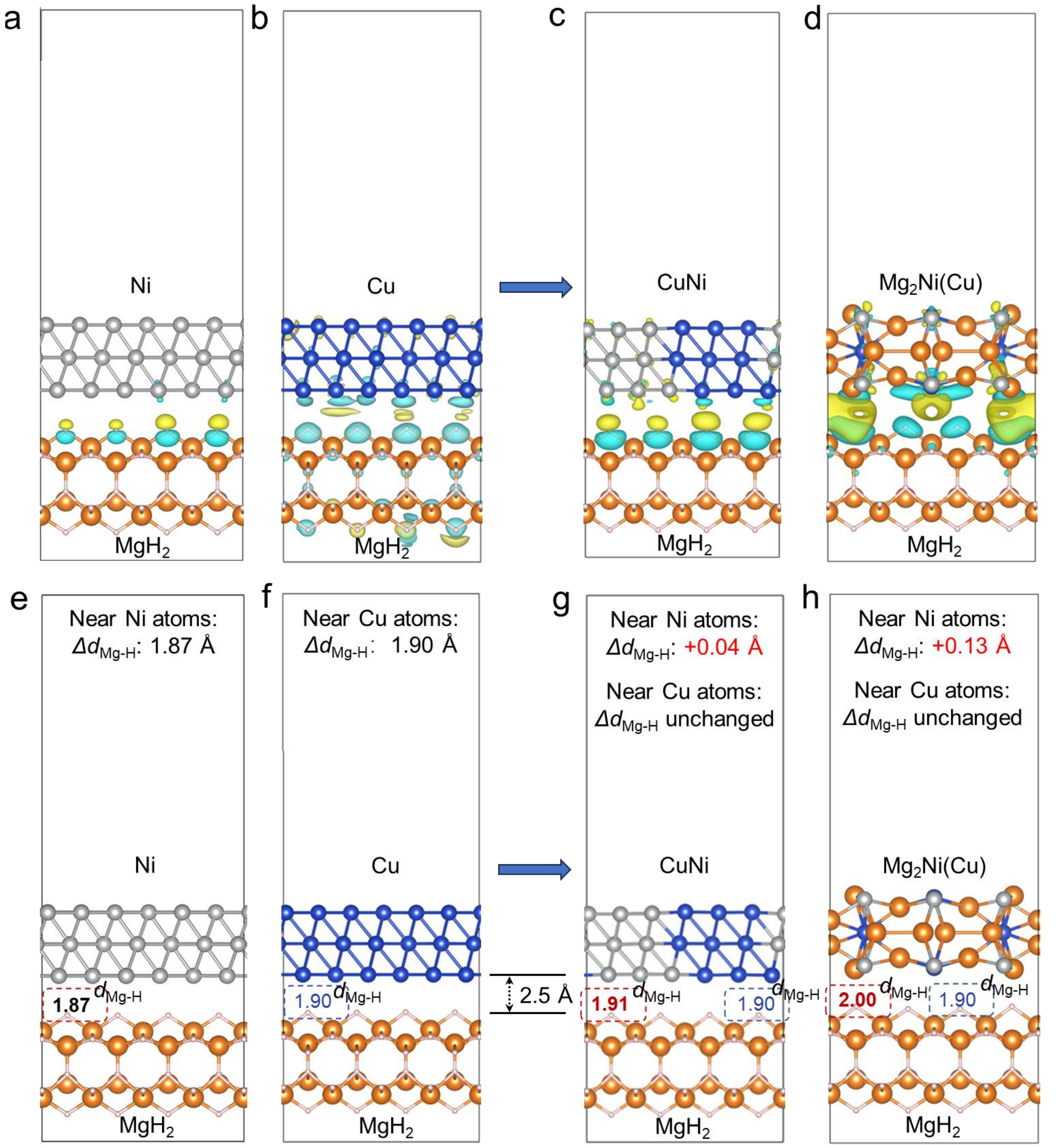

Supplementary Figure 39. The impact of uneven electron distribution around Ni and Cu on the Mg-H bond. The charge density differences of MgH_2 under the catalysis of a) Ni, b) Cu, c) CuNi and d) $Mg_2Ni(Cu)$. The blue area represents electron migration, while the yellow area represents electron aggregation, respectively. The length of Mg-H bond under the catalysis of e) Ni, f) Cu, g) CuNi and h) $Mg_2Ni(Cu)$.

References

1. Ji L, Zhang L, Yang X, Zhu X, Chen L. The remarkably improved hydrogen storage performance of MgH_2 by the synergetic effect of an FeNi/rGO nanocomposite. *Dalton Transactions* **49**, 4146-4154 (2020).
2. Hou Q, Zhang J, Yang X, Ding Z. Ni_3Fe/BC nanocatalysts based on biomass charcoal self-reduction achieves excellent hydrogen storage performance of MgH_2. *Dalton Transactions* **51**, 14960-14969 (2022).
3. Ding Z*, et al.* Improve hydrogen sorption kinetics of MgH_2 by doping carbon-encapsulated iron-nickel nanoparticles. *Journal of Alloys and Compounds* **843**, 156035 (2020).
4. Guemou S*, et al.* Exceptional catalytic effect of novel rGO-supported Ni-Nb nanocomposite on the hydrogen storage properties of MgH_2. *Journal of Materials Science & Technology*, (2023).
5. Huang T*, et al.* Enhancing hydrogen storage properties of MgH_2 through addition of Ni/$CoMoO_4$ nanorods. *Materials Today Energy* **19**, 100613 (2021).
6. Liang H*, et al.* Facile synthesis of nickel-vanadium bimetallic oxide and its catalytic effects on the hydrogen storage properties of magnesium hydride. *International Journal of Hydrogen Energy* **47**, 32969-32980 (2022).
7. Zang J*, et al.* Ni, beyond thermodynamic tuning, maintains the catalytic activity of V species in $Ni_3(VO_4)_2$ doped MgH_2. *Journal of Materials Chemistry A* **9**, 8341-8349 (2021).
8. Zhao Y*, et al.* Enhancing hydrogen storage properties of MgH_2 by core-shell CoNi@C. *Journal of Alloys and Compounds* **862**, 158004 (2021).
9. Huang X*, et al.* Synergistic catalytic activity of porous rod-like $TMTiO_3$ (TM= Ni and Co) for reversible hydrogen storage of magnesium hydride. *The Journal of Physical Chemistry C* **122**, 27973-27982 (2018).
10. Xu N, Wang K, Zhu Y, Zhang Y. PdNi biatomic clusters from metallene unlock record - low onset dehydrogenation temperature for bulk - MgH_2. *Advanced Materials*, 2303173.
11. Chen M*, et al.* Synergy between metallic components of MoNi alloy for catalyzing highly efficient hydrogen storage of MgH_2. *Nano Research* **13**, 2063-2071 (2020).
12. Wang Z*, et al.* In situ formed ultrafine NbTi nanocrystals from a NbTiC solid-solution MXene for hydrogen storage in MgH_2. *Journal of Materials Chemistry A* **7**, 14244-14252 (2019).
13. Zhang L*, et al.* Highly active multivalent multielement catalysts derived from hierarchical porous $TiNb_2O_7$ nanospheres for the reversible hydrogen storage of MgH_2. *Nano Research* **14**, 148-156 (2021).
14. He Z, Zhao J, Lu H. Tunable nonreciprocal reflection and its stability in a non-PT-symmetric plasmonic resonators coupled waveguide systems. *Applied Physics Express* **13**, 012009 (2019).
15. Johnson PB, Christy R-W. Optical constants of the noble metals. *Physical review B* **6**, 4370 (1972).

16. Lee K-S, El-Sayed MA. Gold and silver nanoparticles in sensing and imaging: sensitivity of plasmon response to size, shape, and metal composition. *The Journal of Physical Chemistry B* **110**, 19220-19225 (2006).
17. Sekhon JS, Malik HK, Verma S. DDA simulations of noble metal and alloy nanocubes for tunable optical properties in biological imaging and sensing. *RSC advances* **3**, 15427-15434 (2013).
18. Ma X, Sun H, Wang Y, Wu X, Zhang J. Electronic and optical properties of strained noble metals: Implications for applications based on LSPR. *Nano Energy* **53**, 932-939 (2018).
19. Fang R*, et al.* Transition metal tuned g-C_3N_4 induce highly efficient photocatalytic of ammonia borane to hydrogen evolution and mechanism investigation. *Fuel* **334**, 126707 (2023).
20. Ji G, Wu S, Tian J. High fuel production rate and excellent durability for photothermocatalytic CO_2 reduction achieved via the surface plasma effect of NiCu alloy nanoparticles. *Catalysis Science & Technology* **13**, 2500-2507 (2023).
21. Mayer KM, Hafner JH. Localized surface plasmon resonance sensors. *Chemical reviews* **111**, 3828-3857 (2011).
22. Ding X*, et al.* Surface plasmon resonance enhanced light absorption and photothermal therapy in the second near-infrared window. *Journal of the American Chemical Society* **136**, 15684-15693 (2014).
23. Petryayeva E, Krull UJ. Localized surface plasmon resonance: Nanostructures, bioassays and biosensing-A review. *Analytica chimica acta* **706**, 8-24 (2011).
