# Supplementary material for: Atomic reconstruction for realizing stable solar-driven reversible hydrogen storage of magnesium hydride
Source: Nat Commun. 2024 Apr 1;15:2815. doi: 10.1038/s41467-024-47077-y (PMC10984991; doi:10.1038/s41467-024-47077-y)
Supplement: Supplementary file 4 — Reporting Summary [file 41467_2024_47077_MOESM4_ESM.pdf]

nature portfolio

Corresponding author(s): Professor Guanglin Xia, Professor Fang Fang, Professor Xuebin Yu

Last updated by author(s): Feb 6, 2024

Reporting Summary

Nature Portfolio wishes to improve the reproducibility of the work that we publish. This form provides structure for consistency and transparency in reporting. For further information on Nature Portfolio policies, see our Editorial Policies and the Editorial Policy Checklist.

Statistics

For all statistical analyses, confirm that the following items are present in the figure legend, table legend, main text, or Methods section.

n/a	Confirmed	
☐	☒	The exact sample size (*n*) for each experimental group/condition, given as a discrete number and unit of measurement
☐	☒	A statement on whether measurements were taken from distinct samples or whether the same sample was measured repeatedly
☐	☒	The statistical test(s) used AND whether they are one- or two-sided<br>*Only common tests should be described solely by name; describe more complex techniques in the Methods section.*
☐	☒	A description of all covariates tested
☐	☒	A description of any assumptions or corrections, such as tests of normality and adjustment for multiple comparisons
☐	☒	A full description of the statistical parameters including central tendency (e.g. means) or other basic estimates (e.g. regression coefficient) AND variation (e.g. standard deviation) or associated estimates of uncertainty (e.g. confidence intervals)
☒	☐	For null hypothesis testing, the test statistic (e.g. *F*, *t*, *r*) with confidence intervals, effect sizes, degrees of freedom and *P* value noted<br>*Give P values as exact values whenever suitable.*
☒	☐	For Bayesian analysis, information on the choice of priors and Markov chain Monte Carlo settings
☒	☐	For hierarchical and complex designs, identification of the appropriate level for tests and full reporting of outcomes
☒	☐	Estimates of effect sizes (e.g. Cohen's *d*, Pearson's *r*), indicating how they were calculated

Our web collection on statistics for biologists contains articles on many of the points above.

Software and code

Policy information about availability of computer code

Data collection

1. X-Ray Diffraction (XRD; D8 advance, Bruker AXS, USA) with Cu Kα radiation (1.542 Å) at 40 kV and 40 mA was used to determine the phase compositions. The materials were protected against oxidation from the air by adhesive tape (amorphous with a broad peak around 2θ ≈ 20°).
2. X-ray photoelectron spectroscopy (XPS) results were obtained on a Thermo Scientific K-Alpha system equipped with a dual X-ray source, adopting an Al Kα (1486.6 eV) anode with a hemispherical energy analyzer.
3. Time-of-flight (TOF) powder neutron diffraction (NPD) data were collected from a general-purpose powder diffractometer (GPPD) (90° bank) at the China Spallation Neutron Source (CSNS), Dongguan, China.
4. The morphologies of the synthesized catalysts and MgH2 catalyzed by CuNi with different states were observed by scanning electron microscopy (SEM, JEOL 7500FA, Japan) and transmission electron microscopy (TEM, JEOL JEM-2100F, Japan).
5. The Cu and Ni contents of samples were measured by an inductively coupled plasma source mass spectrometer (ICP-MS) (Agilent ICPOES730).
6. The absorption spectra of samples in the wavelength range of 200-2200 nm were determined using a UV-vis-NIR Spectrophotometer (Lambda 750, Perkin-Elmer, USA).
7. The content of carbon and alloy were analyzed by thermogravimetric analysis (Discovery TGA 550, USA) from room temperature to 800 °C at a rate of 10 °C/min in the air.
8. Temperature-programmed desorption (TPD), isothermal dehydrogenation tests, and corresponding hydrogenation process of the as-prepared samples were conducted on a home-built high-pressure gas sorption apparatus (HPSA-auto).
9. Density functional theory (DFT) calculations were carried out using Vienna ab initio simulation package (VASP) Version 5.4.4

Data analysis

1. VASPKIT Version:1.3.3 VASPKIT aims at providing a powerful and user-friendly interface to perform high throughput analysis of various material properties from the raw calculated data using the widely-used VASP code. The program can be conveniently run under either the interactive 4user interface or command line mode.

2. Neutron diffraction patterns were recorded at room temperature. Rietveld refinements of the NPD patterns were performed using the GSAS software.
3. XPS data was analyzed by Advantage 5.52.
4. TEM data were processed using DigitalMicrograph, and the particle size was measured using Nano Measurer 1.2 software.

For manuscripts utilizing custom algorithms or software that are central to the research but not yet described in published literature, software must be made available to editors and reviewers. We strongly encourage code deposition in a community repository (e.g. GitHub). See the Nature Portfolio guidelines for submitting code & software for further information.

Data

Policy information about availability of data

All manuscripts must include a data availability statement. This statement should provide the following information, where applicable:

- Accession codes, unique identifiers, or web links for publicly available datasets
- A description of any restrictions on data availability
- For clinical datasets or third party data, please ensure that the statement adheres to our policy

The authors confirm that the data supporting the findings of this study are available within the article and/or its supplementary materials. And the data that support the findings of this study are available on request from the corresponding author upon reasonable request.

Research involving human participants, their data, or biological material

Policy information about studies with human participants or human data. See also policy information about sex, gender (identity/presentation), and sexual orientation and race, ethnicity and racism.

Reporting on sex and gender	This work involves research in the fields of chemistry and energy, and does not involve the collection of sex and gender data.
Reporting on race, ethnicity, or other socially relevant groupings	This work involves research in the fields of chemistry and energy and does not involve the collection of data related to race, ethnicity, or other socially relevant groupings.
Population characteristics	The research focuses on chemical samples and does not involve population characteristics information.
Recruitment	The research focuses on chemical samples and does not involve recruitment.
Ethics oversight	This is not relevant in our experiment.

Note that full information on the approval of the study protocol must also be provided in the manuscript.

Field-specific reporting

Please select the one below that is the best fit for your research. If you are not sure, read the appropriate sections before making your selection.

☐ Life sciences ☐ Behavioural & social sciences ☒ Ecological, evolutionary & environmental sciences

For a reference copy of the document with all sections, see nature.com/documents/nr-reporting-summary-flat.pdf

Ecological, evolutionary & environmental sciences study design

All studies must disclose on these points even when the disclosure is negative.

Study description	In this study, a single-component phase of the Mg2Ni(Cu) ternary alloy was designed through atomic reconstruction to achieve the optimal integration of outstanding photothermal and catalytic effects, aiming to realize stable solar-driven hydrogen storage in MgH2. Quantitative data related to the study have been elucidated in the results section, outlining factors and interactions. Hydrogen storage measurements, integral to the study, are extensively detailed in the 'Hydrogen storage measurement' section of the article. The experimental data represent general outcomes from multiple replicated experiments, ensuring reproducibility.
Research sample	In the 'Materials' section, the manufacturers' sources for the various chemicals used in the chemical synthesis have been specified. The sample preparation methods primarily involve the synthesis of CuNi alloys and MgH2 catalyzed by CuNi alloys, and these methods have been extensively detailed in the 'Methods' section of the main text. The focus of the study is on samples prepared using these methods, and the sample preparation procedures demonstrate repeatability. The experimental outcomes represent general results obtained from the application of these methods, exhibiting a high degree of representativeness.
Sampling strategy	For the individual sample discussed in the article, the preparation batches exceed 10, with a cumulative quantity exceeding 5 grams. The amount of sample used for a single performance test ranges between 20-100 mg, and the quantity for characterization varies based on the specific requirements of each characterization method. The sample preparation quantity is sufficiently substantial, and the selection of samples is done randomly across an ample number of samples and different preparation batches. Adhering strictly to the statements in the 'Methods' section, the material's performance exhibits repeatability across different batches. The selection of samples and the quantity prepared are well-suited to ensure robust and reliable results.

Data collection	The methods for material characterization, including the instruments, have been thoroughly detailed in the 'Materials Characterization' section. Data collection was automated through the instruments, and subsequent data processing and reliability verification were conducted by the authors. All authors take responsibility for the authenticity and reliability of the data.
Timing and spatial scale	The data collection period ranges from September 2022 to December 2023. All material performance data were collected at Fudan University in Shanghai, China. The neutron diffraction data were specifically gathered at the China Spallation Neutron Source (CSNS) in Dongguan, China.
Data exclusions	The results of this experiment do not involve any data exclusions.
Reproducibility	The experiments conducted in this study exhibit reproducibility. Instances of experiment failure were attributed to inadequate control of experimental conditions, and relevant lessons learned were summarized. We have endeavored to provide detailed descriptions of experimental procedures in the Methods section, and we believe that adhering to the methods outlined in the article ensures the reproducibility of the experimental results.
Randomization	The sample selection in this experiment was conducted randomly, as detailed in the Sampling Strategy section.
Blinding	The blinding is not relevant to this study.

Did the study involve field work? ☐ Yes ☒ No

Reporting for specific materials, systems and methods

We require information from authors about some types of materials, experimental systems and methods used in many studies. Here, indicate whether each material, system or method listed is relevant to your study. If you are not sure if a list item applies to your research, read the appropriate section before selecting a response.

Materials & experimental systems

n/a	Involved in the study
☒	☐ Antibodies
☒	☐ Eukaryotic cell lines
☒	☐ Palaeontology and archaeology
☒	☐ Animals and other organisms
☒	☐ Clinical data
☒	☐ Dual use research of concern
☒	☐ Plants

Methods

n/a	Involved in the study
☒	☐ ChIP-seq
☒	☐ Flow cytometry
☒	☐ MRI-based neuroimaging

Plants

Seed stocks	This work involves research in the fields of chemistry and energy, and does not involve the seed stocks.
Novel plant genotypes	This work involves research in the fields of chemistry and energy, and does not involve the novel plant genotype.
Authentication	This is not relevant in our experiment.
